# Pancreatic Mass Characterization Using IVIM-DKI MRI and Machine Learning-Based Multi-Parametric Texture Analysis

**DOI:** 10.3390/bioengineering10010083

**Published:** 2023-01-08

**Authors:** Archana Vadiraj Malagi, Sivachander Shivaji, Devasenathipathy Kandasamy, Raju Sharma, Pramod Garg, Siddhartha Datta Gupta, Shivanand Gamanagatti, Amit Mehndiratta

**Affiliations:** 1 Center for Biomedical Engineering, Indian Institute of Technology Delhi, New Delhi 110016, India; 2 Department of Radio-Diagnosis & Interventional Radiology, All India Institute of Medical Sciences, New Delhi 110029, India; 3 Department of Gastroenterology, All India Institute of Medical Sciences, New Delhi 110029, India; 4 Department of Pathology, All India Institute of Medical Sciences, New Delhi 110029, India; 5 Department of Biomedical Engineering, All India Institute of Medical Sciences, New Delhi 110029, India

**Keywords:** artificial neural network, diffusion kurtosis imaging, diffusion-weighted imaging, intravoxel incoherent motion, pancreas, texture analysis, total variation penalty function

## Abstract

Non-invasive characterization of pancreatic masses aids in the management of pancreatic lesions. Intravoxel incoherent motion-diffusion kurtosis imaging (IVIM-DKI) and machine learning-based texture analysis was used to differentiate pancreatic masses such as pancreatic ductal adenocarcinoma (PDAC), pancreatic neuroendocrine tumor (pNET), solid pseudopapillary epithelial neoplasm (SPEN), and mass-forming chronic pancreatitis (MFCP). A total of forty-eight biopsy-proven patients with pancreatic masses were recruited and classified into pNET (*n* = 13), MFCP (*n* = 6), SPEN (*n* = 4), and PDAC (*n* = 25) groups. All patients were scanned for IVIM-DKI sequences acquired with 14 *b*-*values* (0 to 2500 s/mm^2^) on a 1.5T MRI. An IVIM-DKI model with a 3D total variation (TV) penalty function was implemented to estimate the precise IVIM-DKI parametric maps. Texture analysis (TA) of the apparent diffusion coefficient (ADC) and IVIM-DKI parametric map was performed and reduced using the chi-square test. These features were fed to an artificial neural network (ANN) for characterization of pancreatic mass subtypes and validated by 5-fold cross-validation. Receiver operator characteristics (ROC) analyses were used to compute the area under curve (AUC). Perfusion fraction (f) was significantly higher (*p* < 0.05) in pNET than PDAC. The f showed better diagnostic performance for PDAC vs. MFCP with AUC:0.77. Both pseudo-diffusion coefficient (D*) and f for PDAC vs. pNET showed an AUC of 0.73. ADC and diffusion coefficient (D) showed good diagnostic performance for pNET vs. MFCP with AUC: 0.79 and 0.76, respectively. In the TA of PDAC vs. non-PDAC, f and combined IVIM-DKI parameters showed high accuracy ≥ 84.3% and AUC ≥ 0.84. Mean f and combined IVIM-DKI parameters estimated that the IVIM-DKI model with TV texture features has the potential to be helpful in characterizing pancreatic masses.

## 1. Introduction

Pancreatic cancer is a challenging malignancy with a 5-year survival rate of only 10% due to its non-specific symptoms at an early stage [1]. Pancreatic masses are a spectrum of tumors, with pancreatic ductal adenocarcinoma (PDAC) accounting for 90% of all cases and having the worst prognosis. In contrast, pancreatic neuroendocrine tumors (pNETs), mass-forming chronic pancreatitis (MFCP), cystic masses, and solid papillary epithelial neoplasms (SPENs) account for less than 10% of pancreatic tumors with relatively good prognoses [2,3,4]. On imaging, PDAC, MFCP, and pNET may appear similar and distinguishing between these masses with currently available imaging modalities is difficult.

Several diagnostic tools, such as the serum levels of carbohydrate antigen (CA 19-9), have been used to characterize pancreatic masses [5]. However, they have a high false-negative rate, and at the same time benign pancreatic masses can also have high CA 19-9 levels [5]. Fine needle aspiration cytology (FNAC) produces reliable results, but it is susceptible to tumor seeding in the sampling tract and has limitations such as sampling errors [6]. Cystic masses are easily distinguished by their morphological appearance on cross-sectional imaging with CT and MRI [7], however there are phenotypic similarities between other benign entities like MFCP and malignancies like PDAC [8].

Non-invasive assessment of solid pancreatic masses is preferable, and it is feasible with diffusion-weighted imaging (DWI), which is widely recognized for characterization of tumor physiology using apparent diffusion coefficients (ADCs) [9]. The ADC, which is derived from the DWI signal, has been found to capture the change in tumor cellularity [9]. However, ADC is sensitive towards interfering perfusion signals, which becomes significant as the diffusion gradient strength decreases (at lower *b*-*values*) [10,11]. Intravoxel incoherent motion (IVIM) can be useful to study the perfusion information separately from the acquired diffusion data [10]. However, this model assumes a Gaussian distribution of underlying water movement, which may result in estimation errors for diffusion parameters [12]. This is not the case with diffusion kurtosis imaging (DKI), which corrects for the non-Gaussian distribution of water diffusion caused by underlying tissue microstructures [12]. Lu et al. proposed simultaneous modeling of diffusion (molecular diffusion (D)) and perfusion parameters (pseudo-diffusion coefficient (D*) and perfusion fraction (f)) with a kurtosis (k) measure of non-Gaussian distributions of water movements using a hybrid IVIM-DKI model [13]. This model accurately captures the tumor heterogeneity as compared to other DWI models, allowing improved tumor characterization as shown in brain [14], head and neck [13], thyroid [15], and prostate [16] tumors.

The existing research gap in the existing methodology is as follows: simultaneous modelling of all parameters might result in non-physiological heterogeneity in the parametric maps, possibly explaining the lack of wide acceptance of this imaging modality in routine clinical use for a long time. Table 1 shows the advantages and drawbacks of existing techniques. Total variation penalty function (TV) can be used with non-linear least-square optimization; this incorporates a physiologically reasonable spatial constraint into the standard IVIM-DKI model. Previous studies have demonstrated that this method was useful for the removal of non-physiological heterogeneity in the parametric map by iteratively lowering spurious values throughout the parameter estimation [17,18].

Despite developments in CT and MRI technologies, detecting and characterizing pancreatic masses remain difficult. Texture analysis (TA) has shown promise in early diagnosis by collecting imaging characteristics that offer connections between quantitative biomarkers, therapeutic response, and surgical planning [19]. By calculating gray-level patterns and local spatial correlations between patterns and textures in the area, the extracted features give information on inherent characteristics of tumor physiology and heterogeneity [20,21]. Only a few studies have utilized TA on MRI in the characterization of pancreatic masses; it has considerably assisted in the distinction of pancreatic masses [22,23,24,25]. Thus, the main objective of this study was to evaluate the added value of IVIM-DKI analysis with a TV penalty function in texture analysis in the characterization of pancreatic lesions. Second, we evaluated the role of multi-parametric texture analysis of IVIM-DKI for the characterization of pancreatic masses utilizing machine learning-based techniques. The main contributions of this study are:

- We established a novel IVIM-DKI model with a total variation penalty function to achieve improved non-invasive characterization of pancreatic masses.
- Qualitative and mean comparison between IVIM-DKI parametric maps in pancreatic masses such as PDAC, PNET, MFCP, and SPEN were evaluated.
- Cut-off values for each IVIM-DKI parameter were calculated for characterization of pancreatic masses using ROC analysis.
- We attempted to comprehensively investigate texture features of apparent diffusion coefficient (ADC), diffusion coefficient (D), pseudo-diffusion coefficient (D*), perfusion fraction (f), kurtosis (k), and combined texture features of IVIM-DKI parameters with and without ADC.
- Machine learning-based classification of pancreatic masses using ANN was used and compared with other techniques such as decision tree and ensemble.

## 2. Materials and Methods

### 2.1. Study Population

Patients were recruited for this prospective study after approval from the institute ethics committee and informed written consent was obtained from the study participants before enrollment. Patients were recruited and categorized as having a solid or cystic pancreatic mass based on preliminary MRI. A total of seventy-eight patients (*n* = 78) with pancreatic masses were included from May 2017 to June 2019. Enrolment exclusion criteria of this study were patients with: 1. unequivocal pseudocyst; 2. collection in acute/chronic pancreatitis; 3. prior history of treatment/surgery for a pancreatic mass; 4. contraindications to MRI; 5. terminally ill-patients; and 6. refusal of consent. However, only forty-eight patients could be included in this study analysis out of total of seven-eight patients because the imaging workup was incomplete in six patients (*n* = 6), the mass was of extra-pancreatic origin on histopathology analysis in four patients (*n* = 4), the mass was a complication of acute pancreatitis in ten patients (*n* = 10), and ten patients had unequivocal cystic masses (*n* = 10). A final sample size of forty-eight (*n* = 48) was used for further analysis. Pancreatic masses were proven either on histopathology of the surgically resected specimen or on fine needle aspiration cytology (FNAC) of the mass and they were considered as reference standards for thirty-eight (*n* = 38) patients and for remaining ten patients (*n* = 10) imaging was considered as the gold standard. FNAC was performed by using either endoscopic ultrasound (EUS) guidance or transabdominal ultrasound guidance.

### 2.2. MRI Acquisition

All patients underwent MRI on a 1.5T scanner (Achieva, Philips Healthcare, Best, The Netherlands) using a multi-channel phased-array body coil. The MRI sequence protocol used to acquire all the patients’ scans included gradient echo T1-weighted (mDixon) with repetition time (TR): 500 ms and echo time (TE): 2.3 and 4.6 ms, fat-suppressed (FS) T2-weighted (turbo spin-echo (TSE)) was acquired in the axial plane with TR: 1000 ms and TE: 80 ms. Steady-state free precession (SSFP) sequences were acquired in the coronal plane with TR: 500 ms and TE: 50 ms. Thick slab half-Fourier acquisition single-shot turbo spin echo (HASTE) with TR: 8000 ms and TE: 800 ms and driven equilibrium with 90-degree flip-back pulse (RESTORE) with TR: 1000 ms and TE: 6500 ms sequences for heavily T2-weighted Thick slab magnetic resonance cholangiopancreatography (MRCP) sequence (MRCP) were performed. Contrast-enhanced T1-weighted mDixon sequences were acquired in the arterial, pancreatic, and venous phases at 25 s, 45 s, and 70 s, respectively. The patient received 0.2 mL/kg of intravenous gadolinium-based MRI contrast by a pressure injector, followed by a flush of saline.

IVIM-DKI sequences were acquired using free-breathing technique with TR = 1000 ms, TE = 118.7 ms, slice thickness = 6 mm, the field of view (FOV) = 375 × 305 mm, and matrix size = 124 × 100. IVIM-DKI images were acquired at 14 *b*-*values* covering the pancreas in the transverse plane using spin echo-echo planar imaging (SE-EPI) sequence with *b*-*values* (number of averages) of 0(1), 25(1), 50(1), 75(1), 100(1), 150(1), 200(1), 500(2), 800(3), 1000(3), 1250(4), 1500(4), 2000(5), and 2500(7) s/mm^2^. The diffusion sensitizing gradient was applied in three orthogonal directions. Three raw IVIM-DKI images were acquired (one for each diffusion direction) and reconstructed. Then, these three images were averaged in the console to generate one IVIM-DKI image. The total scan time to acquire the IVIM-DKI images was 16 min per patient.

### 2.3. MRI Image Analysis

All IVIM-DKI images were exported from the picture archiving and communication system (PACS) workstation to a personal workstation in digital imaging and communications in medicine (DICOM) format and then converted to neuroimaging informatics technology initiative (NIfTI) format using the dcm2nii tool [26]. Non-linear least-squares optimization and parallel computing with an in-house built toolbox were used for ADC estimation. The ADC parametric map was estimated voxelwise using a monoexponential model with 4 *b*-*values* (i.e., 0, 500, 800, 1000 s/mm^2^) as used in the literature [27,28] and in Equation (1):

(1) $$S/S_{0}=e^{-b\;ADC}\;$$

where ***S*_0_** and ***S*** indicate signal intensities of the images without and with diffusion weighting of b in s/mm^2^.

The IVIM-DKI signal was modelled using two models with 14 *b*-*values* (i.e., 0 to 2500 s/mm^2^): (1) standard model (Equation (2)), where IVIM-DKI parametric maps were obtained by simultaneously fitting for all the four IVIM-DKI parameters (D, D*, f, and k); and (2) IDTV model, where the IVIM-DKI model with TV method was used to estimate IVIM-DKI parametric maps using the open access ivimDKI3Dtvtool_v1_4 toolbox developed in MATLAB (version 9.9, The MathWorks, Inc., Natick, MA, USA) [16]. A trust-region based fit algorithm was used to estimate the IVIM-DKI parameters which allows us to use the bounds for each parameter, D = [0 0.05] mm^2^/s, D* = [0 0.5] mm^2^/s, f = [0 1], and k = [0 3] and was initialized with D = 1.3 × 10^−3^ mm^2^/s, D* = 13 × 10^−3^ mm^2^/s, f = 0.3, and k = 0.7 based on previous studies [29,30].

The IVIM-DKI images at different *b*-*values* for one representative patient are presented in Supplementary Material Figure S1. All IVIM-DKI signals were normalized to the *b-value* = 0 s/mm^2^ signal and Equation (2) shows an association between signal variation and *b*-*values* in the IVIM-DKI sequence [13]:

(2) $$S/S_{0}=fe^{-bD^{*}}+\left(1-f\right)e^{-bD+\frac{1}{6}b^{2}D^{2}k}\;$$

The IDTV model can remove any abrupt change in parameter estimates during the optimization process. The TV penalty function was used to iteratively balance out the estimated parameter [18]. The TV of a 3D parametric map is a L_1_ norm of discrete gradient of the map [16]. The three dimensional-TV (3D-TV) is an adaptive regularization approach that has the benefit of being robust to *b*-*values* combinations and consistently improved parameter estimations at different field strengths [16,31]. The 3D-TV was used in conjunction with the IVIM-DKI model to reconstruct the entire parametric map by considering neighboring voxels in three directions i.e., TV minimization was applied in the entire image at once [16].

### 2.4. Localization of Region of Interest

**Figure 1:**
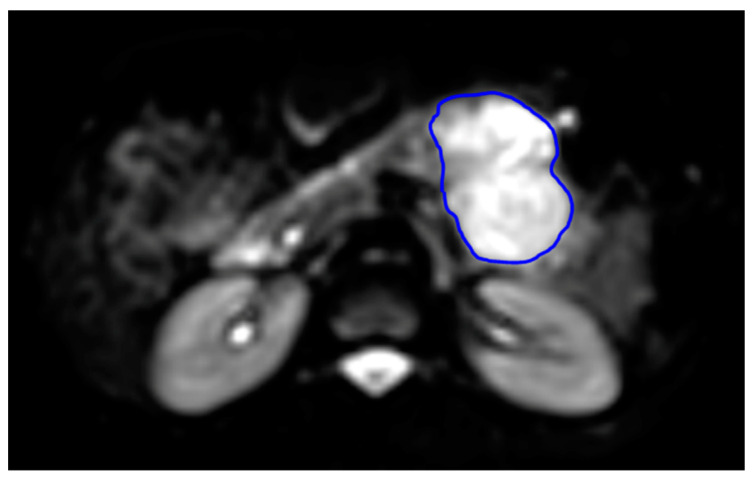
Representation of ROI localization in 27-year-old female patient with pancreatic mass (blue line highlighting the region of SPEN mass) as viewed on IVIM-DKI image at b = 0 s/mm^2^.

A radiologist with more than three years of expertise in abdominal imaging performed the region of interest (ROI) analysis, which was confirmed by another radiologist with more than ten years of experience in abdominal MR imaging and blinded to the reference standard. A free hand ROI was drawn over the lesion to cover the whole mass using MRICron software [26], as shown in Figure 1. The signal intensity of the mass was noted on T1-weighted, T2-weighted, DWI, and ADC images. As shown in Figure 1, the tumor ROI was drawn on the solid margin of the pancreatic tumors as seen on IVIM-DKI at b = 0 s/mm^2^ and ROI was verified by the presence of hyperintensity and hyperintensity on corresponding slices of IVIM-DKI at b = 2500 s/mm^2^ and ADC map, respectively. Mass ROIs were defined on all matching slices of IVIM-DKI images, using ADC maps and conventional sequences such as T2-weighted, contrast enhanced T1-weighted, and DWI images as a reference.

### 2.5. Texture Feature Calculation and Machine Learning-Based Classification

**Figure 2:**
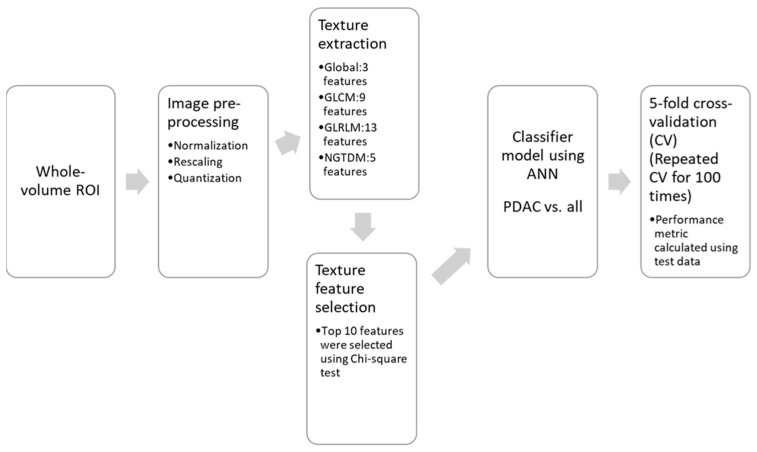
Flowchart representing the machine learning-based classification of pancreatic masses using texture features of ADC and IVIM-DKI parameters.

Figure 2 represents the flowchart of the machine learning-based classification of pancreatic masses using texture features from ADC and IVIM-DKI parameters. Three-dimensional TA was performed for the classification of pancreatic masses using the whole volume ROI which was superimposed onto ADC and IVIM-DKI parametric maps. A total of 30 texture features were extracted from global texture (3 features), Gray-Level Co-occurrence Matrix (GLCM: 9 features), Gray-Level Run-Length Matrix (GLRLM: 13 features), and Neighborhood Gray-Tone Difference Matrix (NGTDM: 5) with 26-voxel connectivity (details of all calculated texture features is presented in the Supplementary Materials, Table S1) using the toolbox developed by Vallière, M. et al. [32]. Prior to TA, normalization and quantization were used to equalize histograms and standardize the intensity range to 64 [32].

The chi-square test of independence was used to select the top ten texture features with the highest feature importance. These top ten features were fed into a 5-layered artificial neural network (ANN) classifier model to differentiate between PDAC and non-PDAC masses. We wanted to investigate the role of an ANN, the most basic form of deep neural network, in classification using IVIM-DKI parametric maps because it has shown potential in screening pancreatic cancer [33]. Bayesian optimization was used to optimize the ANN hyperparameters such as layer output size, activation function, lambda, layer weights, and bias initializer. The Bayesian optimizer tuned the hyperparameters to minimize the cross-validation error of the ANN. Following optimization, the output sizes of each hidden layer were set to (221, 16, 3, 2, 2) using the activation function ‘tanh’, the weights and bias of each layer were started with one using the ‘he’ initializer, and lambda was set to 0.0061374 for regularization of the hyperparameter tuning. These optimized hyperparameters were fed into an ANN architecture that consisted of an input layer containing predictor variables and class labels, ‘tanh’ as the activation function for each hidden layer, and a final layer with ‘softmax’ as the activation function and an output layer that returned the predicted class label. MATLAB was used for the TA and machine learning-based classification (version 9.9, The MathWorks, Inc., Natick, MA, USA).

### 2.6. Statistical Analysis

The patients’ mean age and gender distribution were compared using a two-sample *t*-test and chi-square test. The ADC and IVIM-DKI parameters (D, D*, f, and k) were tested for normality using the Kolmogorov–Smirnov test. The goodness of fit in the standard and IDTV model was assessed using mean adjusted R^2^ in tumor ROI, with a high value of adjusted R^2^ indicating that the model adequately reflects the data. The coefficient of variation (CV: standard deviation of ROI × 100/mean of ROI) of the IVIM-DKI parameters estimated using standard and IDTV models in pancreatic masses was measured to evaluate the precision of model estimations, where low CV indicates precise estimation of parameters by a model. The Wilcoxon signed-rank test was used to determine significant differences between mean adjusted R^2^ of the two comparative models. A Kruskal–Wallis test with a Tukey–Kramer multi-comparison test was used to compute comparison between IVIM-DKI parameters for subtypes of pancreatic masses (PDAC, pNET, MFCP, and SPEN). The diagnostic performance of a IDTV model was assessed using receiver operating characteristics (ROC) analysis with cut-off values, area under the ROC curve (AUC), sensitivity, and specificity.

Important texture features were computed using the chi-square test of independence. The chi-square test of independence was used to determine the relationship between feature variables (texture features derived from ADC or IVIM-DKI parameters) and target variables (class labels: pancreatic masses). If the *p*-value was small, the feature was considered as an important feature and contributed to one of the significant characteristics in classification; otherwise, it was disregarded if the feature had a large *p*-value and was not considered as an important feature. Thus, the top ten texture features were chosen to improve performance and simplify the complexity of the classifier model. Performance metrics were estimated such as accuracy, precision, recall, specificity, accuracy, F1 score, and classification error on test data from each stratified fold of the 5-fold cross-validation (training data: *n* = 38; testing data: *n* = 10) and this cross-validation was repeated 100 times. A test set from each cross-validation was used to evaluate the performance of the ANN in the classification of PDAC vs. non-PDAC. This 5-fold cross-validation was repeated 100 times; thus, the result was 100 different performance metrics. This validation technique ensures that the data of each patient appeared in the test set a total of 100 times. All statistical analyses were performed using MATLAB (version 9.9, The MathWorks, Inc., Natick, MA, USA).

## 3. Results

### 3.1. Patient Population and Tumor Volume

Based on the reference standard, the pancreatic masses were composed of SPENs (*n* = 4; age = 29.5 ± 5.1 years old; female:male = 3:1), MFCP (*n* = 6; age = 46.4 ± 15.1 years old; female:male = 0:6), pNETs (*n* = 13; age = 41.7 ± 13.9 years old; female:male = 4:9), and PDAC (*n* = 25; mean ± SD = 57.9 ± 11 years old; female:male = 2:23). Table 2 contains a summary of the patient’s characteristics. The mean age of patients with PDAC was significantly older (*p* < 0.05) than patients with SPENs and pNETs. Gender differences in pancreatic mass subtypes were significant (*p* < 0.05) between patients with SPEN vs. PDAC and PDAC vs. pNET. No gender differences between patients with and SPEN vs. pNET were observed. Tumor volume size was estimated for each pancreatic mass: SPEN = 52.14 ± 51.77 cm^3^, MFCP = 22.98 ± 17.05 cm^3^, pNET = 8.73 ± 8.02 cm^3^, and PDAC = 33.43 ± 29 cm^3^.

### 3.2. Model Performance in Pancreatic Masses

**Figure 3:**
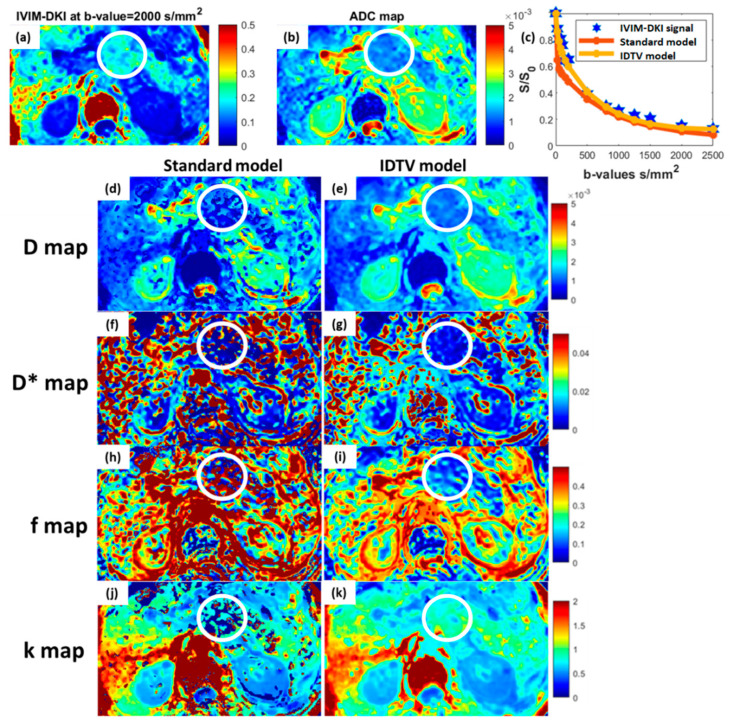
Representative image of a 72-year-old male patient with PDAC demonstrating qualitative comparison between parameter maps estimated using standard (**d**,**f**,**h**,**j**) and IDTV (**e**,**g**,**i**,**k**) models. The encircled zone (white) represents the tumor-affected region, and the IDTV model successfully eliminated noise from the parameter maps when compared to the standard approach and (**c**) accurately curve fitting the mean of the IVIM-DKI signal decay for the tumor region. (**a**) IVIM-DKI image at b = 2000 mm^2^/s appears hyperintense in encircled region and (**b**) hypointense in corresponding region in ADC map. (**k**) In k map, hyperintensity was seen in tumor region, while (**e**,**g**,**i**) hypointensity was observed in D, D*, and f maps. (**d**,**e**) D map, (**f**,**g**) D*map, (**h**,**i**) f map, and (**j**,**k**) k map.

**Figure 4:**
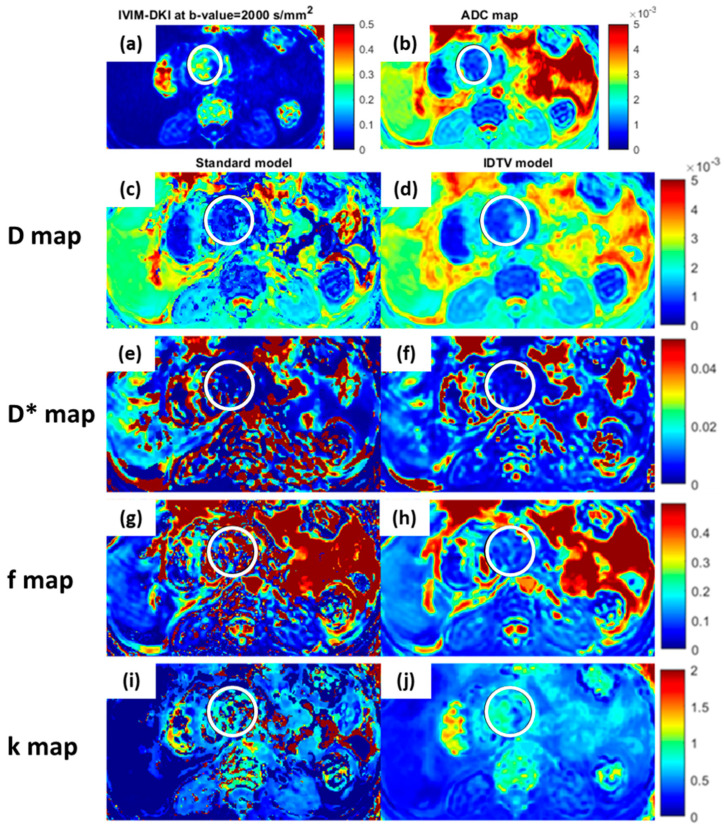
Representative image of a 53-year-old male patient with pancreatic ductal adenocarcinoma (PDAC) demonstrating qualitative comparison between parametric maps estimated using standard (**c**,**e**,**g**,**i**) and IDTV (**d**,**f**,**h**,**j**) models. The encircled zone (white) represents the tumor, and the IDTV model successfully eliminated noise from the parametric maps when compared to the standard model. (**a**) IVIM-DKI image at b = 2000 mm^2^/s appears hyperintense in encircled region and (**b**) hypointense in corresponding region in ADC map. (**j**) In k map, hyperintensity was seen in the tumor region, while (**d**,**f**,**h**) hypointensity was observed in the D, D*, and f maps. (**c**,**d**) D map, (**e**,**f**) D*map, (**g**,**h**) f map, and (**i**,**j**) k map.

Two representative images of IVIM-DKI at high *b-value* = 2000 s/mm^2^, ADC, and IVIM-DKI parameters maps of a 72-year-old male patient with PDAC are presented in Figure 3a,b,d–k and of a 53-year-old male patient with PDAC in Figure 4a–j and were qualitatively compared. The PDAC-affected region appeared to be hypointense in D* (Figure 3g and Figure 4f) and f maps (Figure 3i and Figure 4h), whereas in SPEN, it can be clearly observed that the tumor region appeared hyperintense in k and hypointense in the D, D*, and f maps estimated using the IDTV model as shown in Supplementary Material Figures S2 and S3. In MFCP, the tumor region appeared hyperintense in the f and a hypointense region was observed in D, D*, and k as shown in Supplementary Material Figures S4 and S5. The pNET tumor region appeared to be hyperintense in the D* and f and hypointense in the D and k as shown in Supplementary Material Figures S6 and S7. Additional representative images of seven patients with pancreatic masses are presented in Supplementary Material Figures S2–S8, which show parametric maps from the standard and IDTV models for qualitative comparison.

The goodness of fit was significantly higher (*p* < 0.001) for the IDTV model with R^2^ = 0.95 ± 0.04 compared to the standard model with R^2^ = 0.78 ± 0.16. Figure 3c shows the fitting of the IVIM-DKI signal from one representative patient’s data estimated using the standard and IDTV models. Overall, the IDTV model provided precise estimation of IVIM-DKI parameters with lower CV by 22–64% compared to standard model, except for D* in pancreatic masses (the results are presented in the Supplementary Material, Table S2).

### 3.3. Quantitative Comparison between Subtypes of Pancreatic Masses

**Figure 5:**
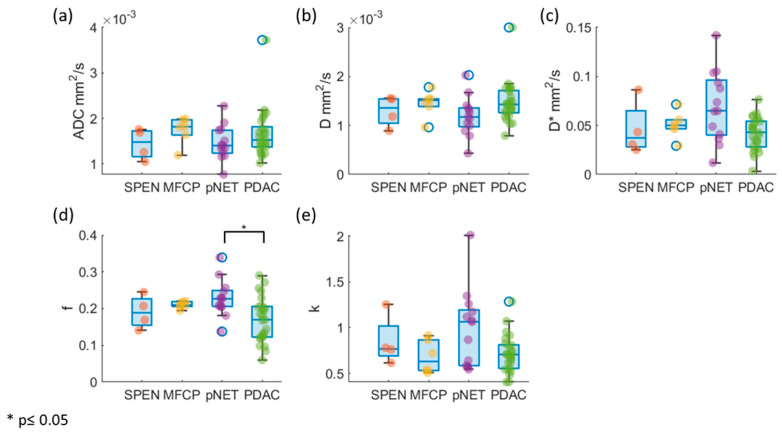
Box and whisker plot showing the distribution of ADC and IVIM-DKI parameters estimated using the IDTV model in PDAC, MFCP, SPEN, and pNET. (**a**–**c**,**e**) ADC, D, D*, and k showed no significant differences in pancreatic masses, and (**d**) f showed significantly higher difference in pNET compared to PDAC.

Figure 5a–e shows the boxplots of the ADC and IVIM-DKI parameters’ distribution in PDAC, SPEN, MFCP, and pNET. ADC, D, D*, and k values did not exhibit any significant differences (*p* > 0.05); however, f values did demonstrate significant differences between pancreatic masses as shown in Table 3. Multiple comparisons between pancreatic masses revealed that the mean f (*p* < 0.05) was considerably higher in the pNET as compared to the PDAC (the results of multiple comparisons can be seen in Supplementary Material, Table S3), as illustrated in Figure 5d.

### 3.4. Differential Diagnosis of Pancreatic Masses Using ROC Analysis

Cut-off values from the ADC and IVIM-DKI parameters were obtained using ROC analysis to differentiate the pancreatic masses such as PDAC, SPEN, MFCP, and pNET, as shown in Table 4. For PDAC vs. MFCP, only the f parameter showed a high AUC of 0.77 with the cut-off value at 0.20496 having an accuracy, sensitivity, and specificity of 77%, 83%, and 76%, respectively. D* and f showed high AUCs of 0.73 with cut-off 63.73 × 10^−3^ mm^2^/s and 0.20424 having an accuracy of 88% and 76%, sensitivity of 63% and 75%, and specificity of 96% and 76%, respectively, to differentiate PDAC from pNET. For pNET vs. MFCP, ADC showed a high AUC of 0.79 with the cut-off 1.58 × 10^−3^ mm^2^/s having an accuracy of 79%, sensitivity of 83%, and specificity of 75%. In addition, the D parameter showed a high AUC of 0.76 with the cut-off value at 1.37 × 10^−3^ mm^2^/s having an accuracy, sensitivity, and specificity of 79%, 83%, and 75%, respectively.

### 3.5. Multi-Parametric Texture Analysis and Machine Learning-Based Classification of Pancreatic Masses

A total of 30 texture features were calculated for individual parameters, ADC, D, D*, f, and k; thus, 150 textural features (30 × 5) were calculated for each mass. The features of all IVIM-DKI parameters were combined to form a 120 (30 features from each parameter, 30 × 4) texture-feature set. Similarly, ADC and IVIM-DKI parametric maps’ texture features were combined to create a total of 150 feature sets. These features were then reduced by selecting only high importance scores which were calculated using the Chi-square test and only the top ten features were selected to reduce the model complexity for PDAC vs. non-PDAC pancreatic masses.

In PDAC vs. non-PDAC, an ANN with all the features of the combined IVIM-DKI parameters showed the highest accuracy of 90.5%, and AUC of 0.92 as shown in Table 5. Classification performance after feature reduction using chi-square decreased by 3–8.5% for combined texture features from IVIM-DKI parameters and individual IVIM-DKI parameters. The top 10 texture features from f showed the highest accuracy of 84.9% and an AUC of 0.85. The combined IVIM-DKI parameters and IVIM-DKI parameters with ADC also performed better with an accuracy of 84.3% and AUC of 0.84. The selected top texture features from the combined IVIM-DKI parameters and IVIM-DKI parameters with ADC were from f, D*, and k. Additionally, decision tree and ensemble were used to characterize PDAC vs. non-PDAC. As shown in Supplementary Table S4, the decision tree performed poorly for PDAC vs. non-PDAC as compared to ensemble and ANN. However, the decision tree showed that the combined texture features of IVIM-DKI parameters after feature reduction showed the highest accuracy and an AUC of 74% and 0.8, respectively. Even the ANN showed a high accuracy and AUC of 84.3% and 0.84 with the combined texture features of IVIM-DKI parameters after feature reduction. Meanwhile, ensemble showed that textural features of the D parameter after feature reduction showed the highest accuracy and an AUC of 91.8% and 0.97, respectively. Commonly selected texture features from multi-parameters were from GLCM (f9), GLRLM (f12, f13, f16, and f21), and NGTDM (f29).

## 4. Discussion

MRI can aid in the differential diagnosis of pancreatic masses which is otherwise a very challenging task. With the assumption of a non-Gaussian distribution of water movement, IVIM-DKI can non-invasively measure diffusion and perfusion parameters to capture the tumor heterogeneity. The efficiency of the novel IVIM-DKI model with a total variation penalty function for characterizing pancreatic mass subtypes was evaluated. f was observed to be useful in distinguishing pancreatic masses. In the differential diagnosis of pancreatic masses, f was found to be a crucial indicator in effectively differentiating PDAC from MFCP and both D* and f for differentiating PDAC from pNET. Further, ADC and D could distinguish pNET from MFCP with good accuracy and AUC. In addition, IVIM-DKI parameters were subjected to textural analysis with textural feature reduction and given to the 5-layer artificial neural network for classification of pancreatic masses. PDAC was distinguished from non-PDAC by texture features of f and combined IVIM-DKI parameters with good accuracy and AUC. IVIM-DKI with the IDTV model was able to remove noise from the parametric maps and produce precise parameter values with low CV. These results demonstrate that the IVIM-DKI model with a spatially constrained optimization method may provide true underlying texture features for successful characterization of pancreatic masses.

A new approach was used to precisely measure IVIM-DKI parameter values to differentiate between pancreatic masses. This IDTV model has been effectively applied in characterization of prostate lesions [16] and patients with lymphoma [34]. The TV penalty function is an adaptive regularization approach that iteratively balances the regularization weight while preserving lesion edge [17,18]. The IDTV model has demonstrated good description of the underlying data in tumor tissues and reduced fitting residuals when estimating the parameters, which was consistent with the findings of Baidya Kayal et al. (2017) [18], who also demonstrated improved parameter estimations using the IVIM model with TV in osteosarcoma [18]. Repeatability was not assessed in this study because we did not obtain repeat scans; however, previous research has shown significant inter-scan agreement and no variability in parameter estimation in healthy tissue volume across time points, proving the repeatability and reproducibility of this new approach in clinical scenarios [35]. Additionally, parametric maps assessed using the IDTV model were found to be less noisy than the standard model [13,16,18]. However, the real clinical impact of these parametric maps estimated using the IDTV model would need multi-centric and multi-rater assessment.

Although the efficacy of IVIM-DKI in pancreatic mass characterization has yet to be completely examined, it provides a quantitative evaluation of perfusion and heterogeneity in the tumor. Mean f was able to characterize PDAC from pNET, where f was high in the latter, which might be attributed to the large amount of desmoplastic stroma and poor tumor vascularity in PDAC [36,37,38,39]. Meanwhile, pNET has a well-defined capillary network and is a hypervascular tumor compared to PDAC [36,37]. ADC, D, D*, and k were unable to distinguish between pancreatic masses, which was similarly demonstrated in previous studies [30,36,37,40,41]. This might be due to the differentiating feature of PDAC being tissue perfusion rather than diffusion [30]. Even when using the IDTV model, D* exhibited a high estimation error, indicating that it cannot be employed as a stand-alone biomarker due to its high sensitivity to signal noise and limited repeatability [36,42].

Additionally, this study estimated cut-off values for further subtyping of pancreatic masses. ADC and D showed good accuracy and AUC for characterizing pNET vs. MFCP. The presence of granulation and fibrosis in MFCP [43] may have contributed to the good diagnostic performance of ADC and D for differentiating pNET from MFCP. The D* and f parameters demonstrated a good degree of accuracy and AUC in discriminating PDAC from pNET; f was also useful for discriminating PDAC from MFCP. However, Klauss et al. (2011) [41] showed that the f parameter had a higher AUC in differentiating between PDAC and MFCP than this study (using a IVIM-DKI model with regularization). The former study used a fixed value of D* derived from the healthy subjects and used it in the model to estimate parameter f; this approach was adopted by Klauss et al. to avoid unreliable parameter estimation (f value) by the simultaneous fitting of all four parameters. However, for patient data, this technique may result in a variation in the estimation of f, as D* has been fixed based on a healthy population. In comparison to their model, the IDTV model does not fix or have prior assumptions for any parameter and estimates all the four parameters concurrently which was also done in previous studies using the same model in prostate cancer [16] and lymphoma [34].

Chronic pancreatitis of the mass-forming type has an imaging appearance like PDAC, leading to misdiagnosis on cross-sectional imaging. On CT perfusion imaging (CTP), blood volume (BV) and blood flow (BF) have been shown to differentiate PDAC from MFCP [44,45]. Perfusion fraction (f) has a significant correlation with CTP parameters such as BV and BF [46,47] and microvessel parameters which are an indicator of tumor angiogenesis in PDAC [47]. This further supports that the IVIM-DKI parameters could be an alternative in patients with compromised kidney function where administration of iodinated or gadolinium-based contrast agents is contraindicated. In comparison to earlier studies, only parameter f demonstrated a greater accuracy AUC for discriminating PDAC from MFCP [36]. While it is widely known that differentiating PDAC from MFCP can be problematic because of the overlapping imaging characteristics, perfusion fraction can be effective in accomplishing this task.

In assessing pancreatic masses, whole-volume TA was also carried out for PDAC vs. other masses. TA can offer information on non-invasively detecting small variations in the intensity of a lesion location and can yield additional information on microscopic tissue heterogeneity [40]. This study used texture features of individual and combined IVIM-DKI parameters and the combination of IVIM-DKI parameters and ADC texture features. PDAC was successfully distinguished from other pancreatic tumors utilizing textural features from f and integrated IVIM-DKI parameters. ADC contributed minimal value to this classification performance for detecting PDAC. The TA of individual IVIM-DKI parameters such as f outperformed other parameters after feature reduction in the classification. Following feature reduction, texture features retrieved from integrated IVIM-DKI parameters largely comprised D*, f, and k features and features of GLCM, GLRLM, and NGTDM methods. The texture features from k performed fairly with good diagnostic performance in detecting PDAC; however, the advantages of the kurtosis parameter have not been widely investigated in the literature [38]. Only a few studies have looked at IVIM parameter texture analysis [23], and none have used higher-order texture analysis of IVIM-DKI parameters such as the NGTDM method. An ANN has been used in differentiation of pancreatic masses using dynamic contrast-enhanced MRI but not with IVIM-DKI parameters [48].

### Limitation and Future Scope

This study has a few limitations including, first, the study cohort was small, and the results from analysis might be biased due to it being a single center study and thus conclusion can be considered as preliminary evidence requiring wider validation. Further, this study needs to be performed with large multicentric data analysis. Second, in this cohort, age and gender were not matched; however, no correlation between age or gender distribution with ADC or IVIM-DKI parametric values were observed. Third, the acquisition time to acquire IVIM-DKI was 16 min, which might cause motion artifacts; hence, future investigations will be required to optimize the *b*-*values* for a shorter acquisition duration. Fourth, the correlation between IVIM-DKI parameters and histopathological markers were not assessed in the current study. Fifth, the IDTV model was not compared with Bayesian techniques since it was outside the scope of this study. Lastly, the texture analysis comparing all possible subtypes of pancreatic masses was not performed because the sample size was insufficient to provide conclusive evidence for subtypes analysis; however, data augmentation techniques were used for this limitation, but results were found to be over-biased and inconclusive due to very small dataset for MFCP and SPEN.

A larger dataset of rare pancreatic cancers such as SPEN and MFCP needs to be acquired so that this can help us in understanding its contribution in characterization of pancreatic masses. Machine learning-based texture analysis may benefit not only in the accurate diagnosis and management of cancer, but also in the virtual reality-based surgical procedure planning [49,50]. This method has the potential to aid surgeons in the navigation of hidden structures with few incisions, such as the precise delineation of dissection planes, such as the pancreatic tumor parenchyma. Automatic segmentation of pancreatic masses can be achieved using deep learning with multi-centric large datasets for accurate delineation of tumors.

## 5. Conclusions

In conclusion, this study demonstrated that the IVIM-DKI model with the spatial regularization optimization approach has a strong potential to be used as an additional paradigm for differential diagnosis of pancreatic masses. Perfusion measures such as D* and f were found to out-perform with accuracies of 88% and 77%, respectively in discriminating pancreatic adenocarcinoma from pancreatic neuroendocrine tumor and mass forming-chronic pancreatitis than the diffusion measures (ADC and D). Non-invasive characterization of pancreatic masses can also largely benefit from whole-volumetric texture analysis of f and combined texture features from IVIM-DKI parameters paired with machine learning-based classification with accuracies of 84.9% and 84.3%, respectively.

## Figures and Tables

**Table 1:** Advantages and disadvantages of existing techniques.

MRI Technique	Advantages	Disadvantages
DWI/ADC	DWI allows for the qualitative and quantitative evaluation of tissue diffusivity without the need for contrast agents. ADC is quantified using a simplistic model and provides quantifiable measures of tumor cellularity.	ADC can be affected by perfusion signals from flowing blood effects that can cause overestimations. ADC becomes more sensitive to tissue microscopic characteristics with higher *b*-*values* due to non-Gaussian water diffusion.
IVIM/D/D*/f	Diffusion and perfusion components of tissue can be evaluated independently.	D* and f can be overestimated due to non-Gaussian water diffusion at high *b*-*values* and long scan times.
DKI/D/k	Accurate representation of water interactions inside tumor.	DKI model cannot eliminate the perfusion effect at low *b*-*values*.
IVIM-DKI/D/D*/f/k	IVIM-DKI incorporates diffusion kurtosis analysis to assess intravoxel incoherent motion in tumor tissues with restricted diffusion.	Low SNR with noisy parametric maps and long scan times.

**Table 2:** Clinical demographics of patients with pancreatic masses.

Pancreatic Masses	No. of Patients	Age (Mean ± SD)	Gender Ratio (F:M)
PDAC	25	57.9 ± 11	2:23
pNET	13	41.7 ± 13.9	4:9
MFCP	6	46.4 ± 15.1	0:6
SPEN	4	29.5 ± 5.1	3:1

PDAC: Pancreatic ductal adenocarcinoma; pNET: Pancreatic neuroendocrine tumor; MFCP: Mass-forming chronic pancreatitis; SPEN: Solid pseudopapillary epithelial neoplasm; F: Female; M: Male.

**Table 3:** Comparison between mean of DWI and IVIM-DKI parameters quantified using new approach in pancreatic masses.

Parameters	PDAC	pNET	MFCP	SPEN	*p*-Value
ADC †	1.7 ± 0.5	1.5 ± 0.4	1.7 ± 0.3	1.4 ± 0.3	0.3
D †	1.5 ± 0.4	1.2 ± 0.4	1.5 ± 0.3	1.3 ± 0.3	0.1
D* †	41.6 ± 16.8	69.5 ± 36.4	50.6 ± 13.7	46.4 ± 27.7	0.07
f	0.17 ± 0.06	0.23 ± 0.05	0.21 ± 0.05	0.19 ± 0.05	0.02
k	0.7 ± 0.2	1 ± 0.4	0.7 ± 0.2	0.9 ± 0.3	0.1

† ADC, D, and D* are expressed in 10^−3^ mm^2^/s; PDAC: Pancreatic ductal adenocarcinoma; pNET: Pancreatic neuroendocrine tumor; MFCP: Mass-forming chronic pancreatitis; SPEN: Solid pseudopapillary epithelial neoplasm; ADC: Apparent diffusion coefficient; D: Diffusion coefficient; D*: Pseudo-diffusion coefficient; f: Perfusion fraction; k: Kurtosis; IVIM-DKI: Intravoxel incoherent motion-diffusion kurtosis imaging.

**Table 4:** ROC analysis of characterizing pancreatic masses using ADC and IVIM-DKI parameters quantified using IDTV model.

Parameters	Threshold	Accuracy %	Sensitivity %	Specificity %	F1_Score %	AUC (CI)
PDAC vs. MFCP						
f	0.20496	77	83	76	59	0.77 (0.59–0.96)
PDAC vs. pNET						
D* †	0.06373	88	63	96	71	0.73 (0.54–0.91)
f	0.20424	76	75	76	60	0.73 (0.54–0.91)
pNET vs. MFCP						
ADC †	0.00158	79	83	75	77	0.79 (0.55–1)
D †	0.00137	79	83	75	77	0.76 (0.51–1)

All AUC values are significant at *p* < 0.05; † ADC and D are expressed in mm^2^/s; AUC: Area under curve; CI: Confidence interval; PDAC: Pancreatic ductal adenocarcinoma; pNET: Pancreatic neuroendocrine tumor; MFCP: Mass-forming chronic pancreatitis; SPEN: Solid pseudopapillary epithelial neoplasm; ADC: Apparent diffusion coefficient; D: Diffusion coefficient; D*: Pseudo-diffusion coefficient; f: Perfusion fraction; k: Kurtosis; IVIM-DKI: Intravoxel incoherent motion-diffusion kurtosis imaging.

**Table 5:** Machine learning-based classification performance of all texture features (30 features) and top ten features selected from ADC, combined, and individual IVIM-DKI parameters, and ADC combined with IVIM-DKI parameters to classify PDAC from Non-PDAC pancreatic masses using an ANN.

PDAC vs. Non-PDAC		Features Selected	Accuracy %	Precision %	Specificity %	F1 Score %	AUC	Classification Error %
ADC	All features (30 features)	All features	82 ± 2.3	81.4 ± 2.0	91.4 ± 1.6	78 ± 2.5	0.81 ± 0.09	18.1 ± 1.9
	Chi-square (Top 10 features)	f27, f30, f14, f6, f5, f4, f18, f7, f28, f9	75 ± 2.9	74.1 ± 2.1	86.9 ± 2.3	69.2 ± 2.9	0.73 ± 0.08	25.1 ± 2.0
IVIM-DKI	All features (30 × 4 features)	All features	90.5 ± 1.7	90.4 ± 1.7	93.6 ± 0.9	89.5 ± 2.1	0.92 ± 0.06	9.5 ± 1.7
	Chi-square (Top 10 features)	f_parameter_f21, D*_parameter_f29, f_parameter_f13, f_parameter_f16, D*_parameter_f5, f_parameter_f9, f_parameter_f29, k_parameter_f12, k_parameter_f9, D_parameter_f9	84.3 ± 1.3	84.2 ± 1.2	89 ± 1.1	82.6 ± 1.5	0.84 ± 0.06	15.7 ± 1.2
ADC with IVIM-DKI	All features (30 × 5 features)	All features	90.7 ± 1.0	90.6 ± 1.0	93.8 ± 1.2	89.7 ± 1.2	0.92 ± 0.07	9.3 ± 1
	Chi-square (Top 10 features)	f_parameter_f21, D*_parameter_f29, f_parameter_f13, f_parameter_f16, D*_parameter_f5, f_parameter_f9, f_parameter_f29, k_parameter_f12, k_parameter_f9, D_parameter_f9	84.3 ± 1.3	84.2 ± 1.2	89.0 ± 1.1	82.6 ± 1.5	0.84 ± 0.06	15.7 ± 1.2
D	All features (30 features)	All features	79.5 ± 2.1	78.8 ± 1.7	89.7 ± 1.6	74.8 ± 2.1	0.78 ± 0.09	20.6 ± 1.7
	Chi-square (Top 10 features)	f9, f1, f27, f25, f10, f17, f19, f13, f16, f24	73.6 ± 1.9	72.6 ± 1.6	87.4 ± 1	66.7 ± 2.2	0.71 ± 0.09	26.6 ± 1.5
D*	All features (30 features)	All features	87.8 ± 1.0	87.5 ± 0.9	92.6 ± 1.0	85.9 ± 0.9	0.89 ± 0.07	12.3 ± 0.9
	Chi-square (Top 10 features)	f29, f5, f22, f27, f26, f25, f30, f11, f2, f1	81.8 ± 1.8	81.3 ± 1.2	90 ± 1.6	78.3 ± 1.4	0.80 ± 0.08	18.2 ± 1.1
f	All features (30 features)	All features	90.3 ± 1.8	90.2 ± 1.8	93.2 ± 1.4	89.4 ± 2.0	0.90 ± 0.05	9.7 ± 1.7
	Chi-square (Top 10 features)	f21, f13, f16, f9, f29, f12, f2, f1, f23, f7	84.9 ± 1.4	85.1 ± 1.5	88.7 ± 1.1	83.8 ± 1.5	0.85 ± 0.06	15.0 ± 1.5
k	All features (30 features)	All features	85.9 ± 2.0	85.6 ± 1.6	91.9 ± 2.1	83.5 ± 1.6	0.85 ± 0.07	14.2 ± 1.6
	Chi-square (Top 10 features)	f12, f9, f18, f20, f1, f4, f22, f28, f2, f19	81.2 ± 0.8	80.8 ± 1.0	89.1 ± 1.2	77.8 ± 1.7	0.80 ± 0.08	18.9 ± 0.9

PDAC: Pancreatic ductal adenocarcinoma; AUC: Area under curve; ADC: Apparent diffusion coefficient; D: Diffusion coefficient; D*: Pseudo-diffusion coefficient; f: Perfusion fraction; k: Kurtosis; IVIM-DKI: Intravoxel incoherent motion-diffusion kurtosis imaging.

## Data Availability

The data that support the findings of this study are available from the corresponding author (A.M.) upon reasonable request.

## Supplementary Materials

The following supporting information can be downloaded at: https://www.mdpi.com/article/10.3390/bioengineering10010083/s1, Figure S1: 1 to 14: DWI trace images of a representative patient from IVIM-DKI images at 14 *b*-*values* from 0 to 2500 s/mm^2^ (0, 25, 50, 75, 100, 150, 200, 500, 800, 1000, 1250, 1500, 2000, 2500 s/mm^2^); Figure S2: Representative image of a 28-year-old female patient with solid papillary epithelial neoplasm (SPEN) with qualitative comparison between parameter maps (D, D*, f, and k maps) estimated using standard and IDTV models; Figure S3: Representative image of a 26-year-old female patient with solid papillary epithelial neoplasm (SPEN) with qualitative comparison between parameter maps (D, D*, f, and k maps) estimated using standard and IDTV models; Figure S4: Representative image of a 63-year-old male patient with mass-forming chronic pancreatitis (MFCP) with qualitative comparison between parameter maps (D, D*, f, and k maps) estimated using standard and IDTV models; Figure S5: Representative image of a 60-year-old male patient with mass-forming chronic pancreatitis (MFCP) with qualitative comparison between parameter maps (D, D*, f, and k maps) estimated using standard and IDTV models; Figure S6: Representative image of a 43-year-old male patient with pancreatic neuroendocrine tumor (PNET) with qualitative comparison between parameter maps (D, D*, f, and k maps) estimated using standard and IDTV models; Figure S7: Representative image of a 41-year-old male patient with pancreatic neuroendocrine tumor (PNET) with qualitative comparison between parameter maps (D, D*, f, and k maps) estimated using standard and IDTV models; Figure S8: Representative image of a 33-year-old male patient with pancreatic ductal adenocarcinomas (PDAC) with qualitative comparison between parameter maps (D, D*, f, and k maps) estimated using standard and IDTV models; Table S1: Texture features extracted from Global, GLCM, GLRLM, and NGDZM; Table S2: Coefficient of variation (CV: standard deviation of ROI × 100/mean of ROI) of IVIM-DKI parameters estimated using standard and IDTV models in pancreatic masses; Table S3: *p*-values obtained from Tukey–Kramer multiple comparison test between ADC and IVIM-DKI parameters values estimated using IDTV model in pancreatic masses; Table S4: Machine learning-based classification performance of all texture features (30 features) and top ten features selected from ADC, combined, individual IVIM-DKI parameters, and ADC combined with IVIM-DKI parameters to classify PDAC from non-PDAC pancreatic masses using decision tree and ensemble.

## References

[1] Siegel R.L., Miller K.D., Fuchs H.E., Jemal A. (2022). Cancer statistics. CA Cancer J. Clin..

[2] Orth M., Metzger P., Gerum S., Mayerle J., Schneider G., Belka C., Schnurr M., Lauber K. (2019). Pancreatic ductal adenocarcinoma: Biological hallmarks, current status, and future perspectives of combined modality treatment approaches. Radiat. Oncol..

[3] Ehehalt F., Saeger H.D., Schmidt C.M., Grützmann R. (2009). Neuroendocrine Tumors of the Pancreas. Oncology.

[4] Klein A.P. (2021). Pancreatic cancer epidemiology: Understanding the role of lifestyle and inherited risk factors. Nat. Rev. Gastroenterol. Hepatol..

[5] Ballehaninna U.K., Chamberlain R.S. (2011). Serum CA 19-9 as a Biomarker for Pancreatic Cancer—A Comprehensive Review. Indian J. Surg. Oncol..

[6] Chang D., Nguyen N.Q., Merrett N., Dixson H., Leong R., Biankin A. (2009). Role of endoscopic ultrasound in pancreatic cancer. Expert Rev. Gastroenterol. Hepatol..

[7] Garcea G., Ong S., Rajesh A., Neal C., Pollard C., Berry D., Dennison A. (2008). Cystic Lesions of the Pancreas. Pancreatology.

[8] Kang J.D., Clarke S.E., Costa A.F. (2020). Factors associated with missed and misinterpreted cases of pancreatic ductal adenocarcinoma. Eur. Radiol..

[9] Wang Y., Chen Z.E., Yaghmai V., Nikolaidis P., McCarthy R.J., Merrick L., Miller F.H. (2011). Diffusion-weighted MR imaging in pancreatic endocrine tumors correlated with histopathologic characteristics. J. Magn. Reson. Imaging.

[10] Le Bihan D., Breton E., Lallemand D., Aubin M.L., Vignaud J., Laval-Jeantet M. (1988). Separation of diffusion and perfusion in intravoxel incoherent motion MR imaging. Radiology.

[11] Ye C., Xu D., Qin Y., Wang L., Wang R., Li W., Kuai Z., Zhu Y. (2019). Estimation of intravoxel incoherent motion parameters using low *b*-*values*. PLoS ONE.

[12] Jensen J.H., Helpern J.A., Ramani A., Lu H., Kaczynski K. (2005). Diffusional kurtosis imaging: The quantification of non-gaussian water diffusion by means of magnetic resonance imaging. Magn. Reson. Med..

[13] Lu Y., Jansen J.F., Mazaheri Y., Stambuk H.E., Koutcher J.A., Shukla-Dave A. (2012). Extension of the intravoxel incoherent motion model to non-gaussian diffusion in head and neck cancer. J. Magn. Reson. Imaging.

[14] Wu W.-C., Yang S.-C., Chen Y.-F., Tseng H.-M., My P.-C. (2017). Simultaneous assessment of cerebral blood volume and diffusion heterogeneity using hybrid IVIM and DK MR imaging: Initial experience with brain tumors. Eur. Radiol..

[15] Núñez D.A., Lu Y., Paudyal R., Hatzoglou V., Moreira A.L., Oh J.H., Stambuk H.E., Mazaheri Y., Gonen M., Ghossein R.A. (2019). Quantitative Non-Gaussian Intravoxel Incoherent Motion Diffusion-Weighted Imaging Metrics and Surgical Pathology for Stratifying Tumor Aggressiveness in Papillary Thyroid Carcinomas. Tomography.

[16] Malagi A.V., Netaji A., Kumar V., Kayal E.B., Khare K., Das C.J., Calamante F., Mehndiratta A. (2021). IVIM-DKI for differentiation between prostate cancer and benign prostatic hyperplasia: Comparison of 1.5 T vs. 3 T MRI. Magn. Reson. Mater. Phys. Biol. Med..

[17] Rudin L.I., Osher S., Fatemi E. (1992). Nonlinear total variation based noise removal algorithms. Phys. D Nonlinear Phenom..

[18] Kayal E.B., Kandasamy D., Khare K., Alampally J.T., Bakhshi S., Sharma R., Mehndiratta A. (2017). Quantitative Analysis of Intravoxel Incoherent Motion (IVIM) Diffusion MRI using Total Variation and Huber Penalty Function. Med. Phys..

[19] Khalvati F., Zhang Y., Baig S., Lobo-Mueller E.M., Karanicolas P., Gallinger S., Haider M.A. (2019). Prognostic Value of CT Radiomic Features in Resectable Pancreatic Ductal Adenocarcinoma. Sci. Rep..

[20] Larroza A., Bodí V., Moratal D. (2016). Texture Analysis in Magnetic Resonance Imaging: Review and Considerations for Future Applications.

[21] Liang L., Ding Y., Yu Y., Liu K., Rao S., Ge Y., Zeng M. (2021). Whole-tumour evaluation with MRI and radiomics features to predict the efficacy of S-1 for adjuvant chemotherapy in postoperative pancreatic cancer patients: A pilot study. BMC Med. Imaging.

[22] Deng Y., Ming B., Zhou T., Wu J.-L., Chen Y., Liu P., Zhang J., Zhang S.-Y., Chen T.-W., Zhang X.-M. (2021). Radiomics Model Based on MR Images to Discriminate Pancreatic Ductal Adenocarcinoma and Mass-Forming Chronic Pancreatitis Lesions. Front. Oncol..

[23] Shi Y.-J., Zhu H.-T., Liu Y.-L., Wei Y.-Y., Qin X.-B., Zhang X.-Y., Li X.-T., Sun Y.-S. (2020). Radiomics Analysis Based on Diffusion Kurtosis Imaging and T2 Weighted Imaging for Differentiation of Pancreatic Neuroendocrine Tumors from Solid Pseudopapillary Tumors. Front. Oncol..

[24] Yingwei W., Xinghua Z., Botao W., Ye W., MengQi L., Haiyi W., Huiyi Y., Zhiye C., Wang Y.-W., Zhang X.-H. (2019). Value of Texture Analysis of Intravoxel Incoherent Motion Parameters in Differential Diagnosis of Pancreatic Neuroendocrine Tumor and Pancreatic Adenocarcinoma. Chin. Med. Sci. J..

[25] Abunahel B.M., Pontre B., Kumar H., Petrov M.S. (2021). Pancreas image mining: A systematic review of radiomics. Eur. Radiol..

[26] Rorden C., Brett M. (2000). Stereotaxic Display of Brain Lesions. Behav. Neurol..

[27] Miller F.H., Wang Y., McCarthy R.J., Yaghmai V., Merrick L., Larson A., Berggruen S., Casalino D.D., Nikolaidis P. (2010). Utility of Diffusion-Weighted MRI in Characterization of Adrenal Lesions. Am. J. Roentgenol..

[28] Rosenkrantz A.B., Oei M., Babb J.S., Ba B.E.N., Taouli B. (2011). Diffusion-weighted imaging of the abdomen at 3.0 Tesla: Image quality and apparent diffusion coefficient reproducibility compared with 1.5 Tesla. J. Magn. Reson. Imaging.

[29] Lemke A., Laun F.B., Klau M., Re T.J., Simon D., Delorme S., Schad L.R., Stieltjes B. (2009). Differentiation of Pancreas Carcinoma from Healthy Pancreatic Tissue Using Multiple *b*-*Values*. Investig. Radiol..

[30] Kang K.M., Lee J.M., Yoon J.H., Kiefer B., Han J.K., Choi B.I. (2014). Intravoxel Incoherent Motion Diffusion-weighted MR Imaging for Characterization of Focal Pancreatic Lesions. Radiology.

[31] Malagi A.V., Das C.J., Khare K., Calamante F., Mehndiratta A. (2019). Effect of combination and number of *b values* in IVIM analysis with post-processing methodology: Simulation and clinical study. Magn. Reson. Mater. Phys. Biol. Med..

[32] Vallières M., Kay-Rivest E., Perrin L.J., Liem X., Furstoss C., Aerts H.J.W.L., Khaouam N., Nguyen-Tan P.F., Wang C.-S., Sultanem K. (2017). Radiomics strategies for risk assessment of tumour failure in head-and-neck cancer. Sci. Rep..

[33] Pereira S.P., Oldfield L., Ney A., Hart P.A., Keane M.G., Pandol S.J., Li D., Greenhalf W., Jeon C.Y., Koay E.J. (2020). Early detection of pancreatic cancer. Lancet Gastroenterol. Hepatol..

[34] Malagi A.V., Kandasamy D., Khare K., Pushpam D., Kumar R., Bakhshi S., Mehndiratta A. Qualitative and quantitative comparison between IVIM-DKI and PET/CT imaging in lymphoma. Proceedings of the 29nd Annual Meeting of ISMRM 2021.

[35] Baidya Kayal E., Khare K., Sharma R., Bakhshi S., Kandasamy D., Mehndiratta A. Evaluating Reproducibility and Repeatability of Penalty Function Based Methods for Quantitative Intravoxel Incoherent Motion Analysis. Proceedings of the 31st Joint Annual Meeting of ISMRM-ESMRMB 2022.

[36] Kim B., Lee S.S., Sung Y.S., Cheong H., Byun J.H., Kim H.J., Kim J.H. (2017). Intravoxel incoherent motion diffusion-weighted imaging of the pancreas: Characterization of benign and malignant pancreatic pathologies. J. Magn. Reson. Imaging.

[37] De Robertis R., Cardobi N., Ortolani S., Martini P.T., Stemmer A., Grimm R., Gobbo S., Butturini G., D’Onofrio M. (2019). Intravoxel incoherent motion diffusion-weighted MR imaging of solid pancreatic masses: Reliability and usefulness for characterization. Abdom. Imaging.

[38] Mayer P., Jiang Y., Kuder T.A., Bergmann F., Khristenko E., Steinle V., Kaiser J., Hackert T., Kauczor H.-U., Klauß M. (2020). Diffusion Kurtosis Imaging—A Superior Approach to Assess Tumor–Stroma Ratio in Pancreatic Ductal Adenocarcinoma. Cancers.

[39] Li J., Liang L., Yu H., Shen Y., Hu Y., Hu D., Tang H., Li Z. (2018). Whole-tumor histogram analysis of non-Gaussian distribution DWI parameters to differentiation of pancreatic neuroendocrine tumors from pancreatic ductal adenocarcinomas. Magn. Reson. Imaging.

[40] Granata V., Fusco R., Sansone M., Grassi R., Maio F., Palaia R., Tatangelo F., Botti G., Grimm R., Curley S. (2020). Magnetic resonance imaging in the assessment of pancreatic cancer with quantitative parameter extraction by means of dynamic contrast-enhanced magnetic resonance imaging, diffusion kurtosis imaging and intravoxel incoherent motion diffusion-weighted imaging. Ther. Adv. Gastroenterol..

[41] Klau M., Lemke A., Grünberg K., Simon D., Re T.J., Wente M.N., Laun F.B., Kauczor H.-U., Delorme S., Grenacher L. (2011). Intravoxel Incoherent Motion MRI for the Differentiation Between Mass Forming Chronic Pancreatitis and Pancreatic Carcinoma. Investig. Radiol..

[42] Lee Y., Lee S.S., Kim N., Kim E., Kim Y.J., Yun S.-C., Kuhn B., Kim I.S., Park S.H., Kim S.Y. (2014). Intravoxel Incoherent Motion Diffusion-weighted MR Imaging of the Liver: Effect of Triggering Methods on Regional Variability and Measurement Repeatability of Quantitative Parameters. Radiology.

[43] Suda K., Takase M., Fukumura Y., Kashiwagi S. (2007). Pathology of autoimmune pancreatitis and tumor-forming pancreatitis. J. Gastroenterol..

[44] Yadav A.K., Sharma R., Kandasamy D., Pradhan R.K., Garg P.K., Bhalla A.S., Gamanagatti S., Srivastava D.N., Sahni P., Upadhyay A.D. (2016). Perfusion CT—Can it resolve the pancreatic carcinoma versus mass forming chronic pancreatitis conundrum?. Pancreatology.

[45] Ren S., Zhang J., Chen J., Cui W., Zhao R., Qiu W., Duan S., Chen R., Chen X., Wang Z. (2019). Evaluation of Texture Analysis for the Differential Diagnosis of Mass-Forming Pancreatitis from Pancreatic Ductal Adenocarcinoma on Contrast-Enhanced CT Images. Front. Oncol..

[46] Federau C., O’Brien K., Meuli R., Hagmann P., Maeder P. (2014). Measuring brain perfusion with intravoxel incoherent motion (IVIM): Initial clinical experience. J. Magn. Reson. Imaging.

[47] Mayer P., Fritz F., Koell M., Skornitzke S., Bergmann F., Gaida M.M., Hackert T., Maier-Hein K., Laun F.B., Kauczor H.-U. (2021). Assessment of tissue perfusion of pancreatic cancer as potential imaging biomarker by means of Intravoxel incoherent motion MRI and CT perfusion: Correlation with histological microvessel density as ground truth. Cancer Imaging.

[48] Gao X., Wang X. (2020). Performance of deep learning for differentiating pancreatic diseases on contrast-enhanced magnetic resonance imaging: A preliminary study. Diagn. Interv. Imaging.

[49] Montemurro N., Condino S., Carbone M., Cattari N., D’Amato R., Cutolo F., Ferrari V. (2022). Brain Tumor and Augmented Reality: New Technologies for the Future. Int. J. Environ. Res. Public Health.

[50] Lin J., Tao H., Wang Z., Chen R., Chen Y., Lin W., Li B., Fang C., Yang J. (2022). Augmented reality navigation facilitates laparoscopic removal of foreign body in the pancreas that cause chronic complications. Surg. Endosc..

