# Supplementary material for: Pancreatic Mass Characterization Using IVIM-DKI MRI and Machine Learning-Based Multi-Parametric Texture Analysis
Source: Bioengineering (Basel). 2023 Jan 8;10(1):83. doi: 10.3390/bioengineering10010083 (PMC9854749; doi:10.3390/bioengineering10010083)
Supplement: Supplementary file 1 [file bioengineering-10-00083-s001.zip › bioengineering-2139602-supplementary.pdf]

Supplementary materials

Table S1: Texture features extracted from Global, GLCM, GLRLM, and NGDZM.

Model	Features extracted
**Global (f1 – f3)**	Variance, skewness, kurtosis
**Gray−Level Co−occurrence Matrix, GLCM (f4 – f12)**	Energy, Contrast, Entropy, Homogeneity, Correlation, Sum Average, Variance, Dissimilarity, and AutoCorrelation
**Gray−Level Run−Length Matrix, GLRLM (f13 – f25)**	Short runs emphasis (SRE), long runs emphasis (LRE), grey level nonuniformity (GLN), run−length nonuniformity (RLN), run percentage (RP), low grey level runs emphasis (LGRE), high grey level runs emphasis (HGRE), short−run low grey level emphasis (SRLGE), short−run high grey level emphasis (SRHGE), long−run low grey level emphasis (LRLGE), long−run high grey level emphasis (LRHGE), Gray−Level Variance (GLV), and Run length variance (RLV)
**Neighborhood Gray−Tone Difference Matrix (NGTDM) texture features, NGTDM (f26 – f30)**	Coarseness, Contrast, Busyness, Complexity, Strength

Table S2: Coefficient of variation (CV: standard deviation of ROI x 100/mean of ROI) of IVIM−DKI parameters estimated using standard and IDTV models in pancreatic masses.

IVIM−DKI parameters	Standard model	IDTV model	P−value
**D**	48±13	32±8	<0.001
**D***	150±63	146±43	0.45
**f**	85±25	64±17	<0.001
**k**	98±36	34±14	<0.001

D: Diffusion coefficient; D*: Pseudo−diffusion coefficient; f: Perfusion fraction; k:**Kurtosis; IVIM−DKI: Intravoxel Incoherent motion−diffusion kurtosis imaging;**

Table S3: P−values obtained from Tukey−Kramer Multiple comparison test between ADC and IVIM−DKI parameters values estimated using IDTV model in pancreatic masses.

DWI parameters	Pancreatic masses	pNET	MFCP	SPEN
**ADC**	PDAC	0.65	0.73	0.86
	pNET	−	0.30	1
	MFCP	−	−	0.51
**D**	PDAC	0.14	0.98	0.93
	pNET	−	0.27	0.88
	MFCP	−	−	0.88
**D***	PDAC	0.053	0.73	1
	pNET	−	0.84	0.45
	MFCP	−	−	0.90
**f**	PDAC	0.013	0.28	0.91
	pNET	−	0.96	0.63
	MFCP	−	−	0.90
**k**	PDAC	0.19	0.95	0.76
	pNET	−	0.25	0.99
	MFCP	−	−	0.65

ADC: Apparent diffusion coefficient; D: Diffusion coefficient; D*: Pseudo−diffusion coefficient; DWI: Diffusion−weighted imaging; f: Perfusion fraction; k: kurtosis; PDAC: Pancreatic ductal adenocarcinoma; pNET: Pancreatic neuroendocrine tumor; MFCP: Mass−forming chronic pancreatitis; SPEN: Solid pseudopapillary epithelial neoplasm; D*: Pseudo−diffusion coefficient; f: Perfusion fraction;

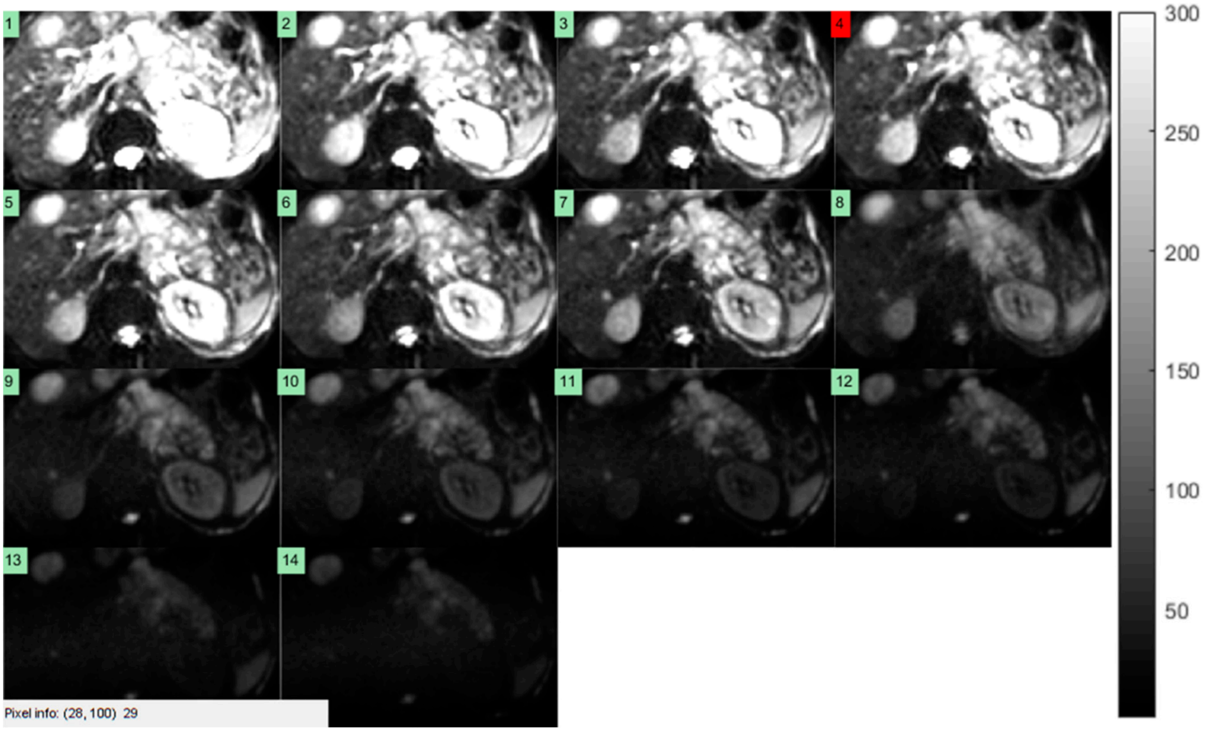

Figure S1: 1 to 14: DWI trace images of a representative patient from IVIM−DKI images at 14 b−values from 0 to 2500 s/mm^2 (0,25,50,75,100,150,200,500,800,1000,1250,1500,2000,2500 s/mm^2).

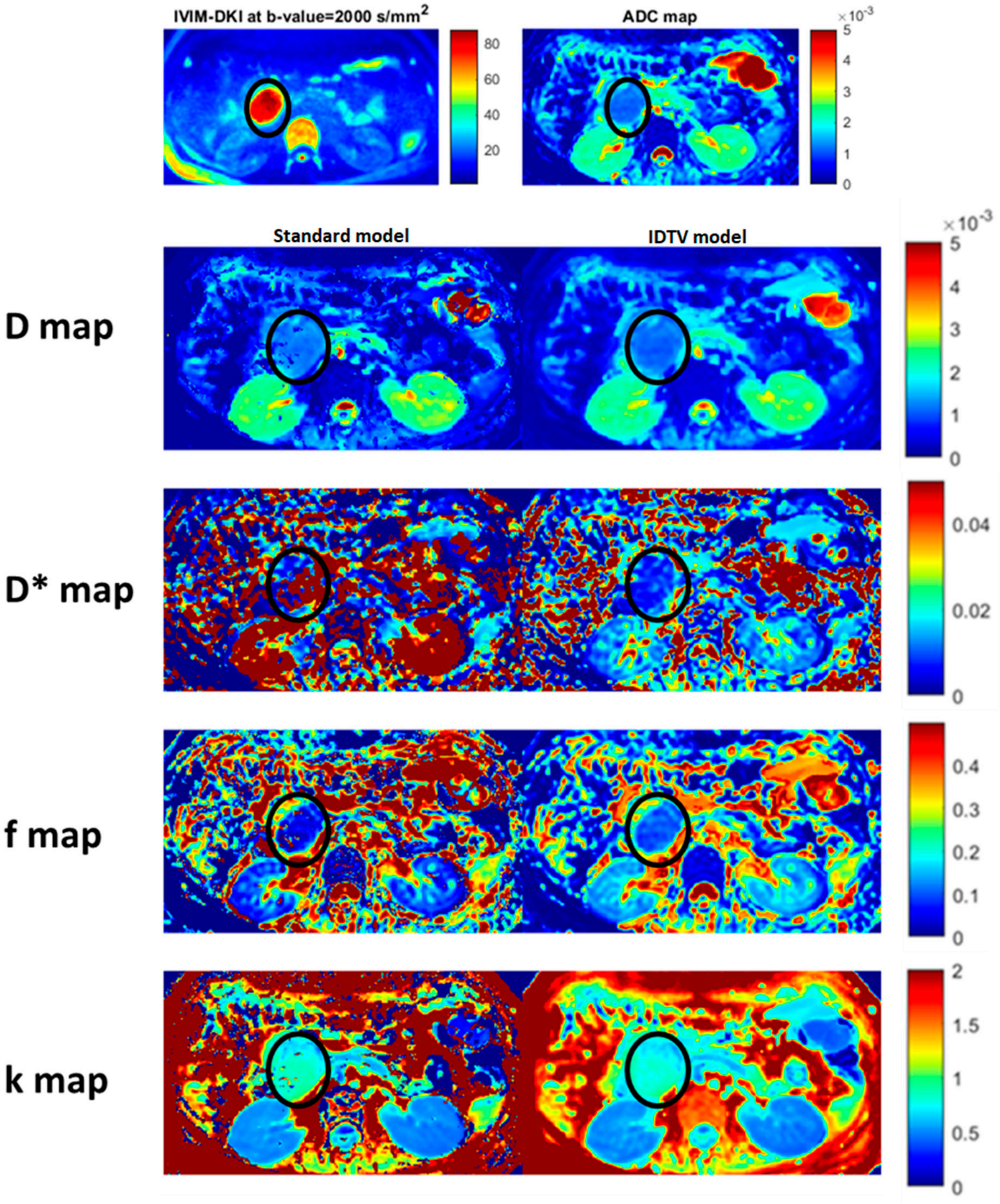

Figure S2: Representative image of a 28−years−old female patient with solid papillary epithelial neoplasm (SPEN); where qualitative comparison between parameter maps (D, D*, f and k maps) estimated using standard and IDTV model.

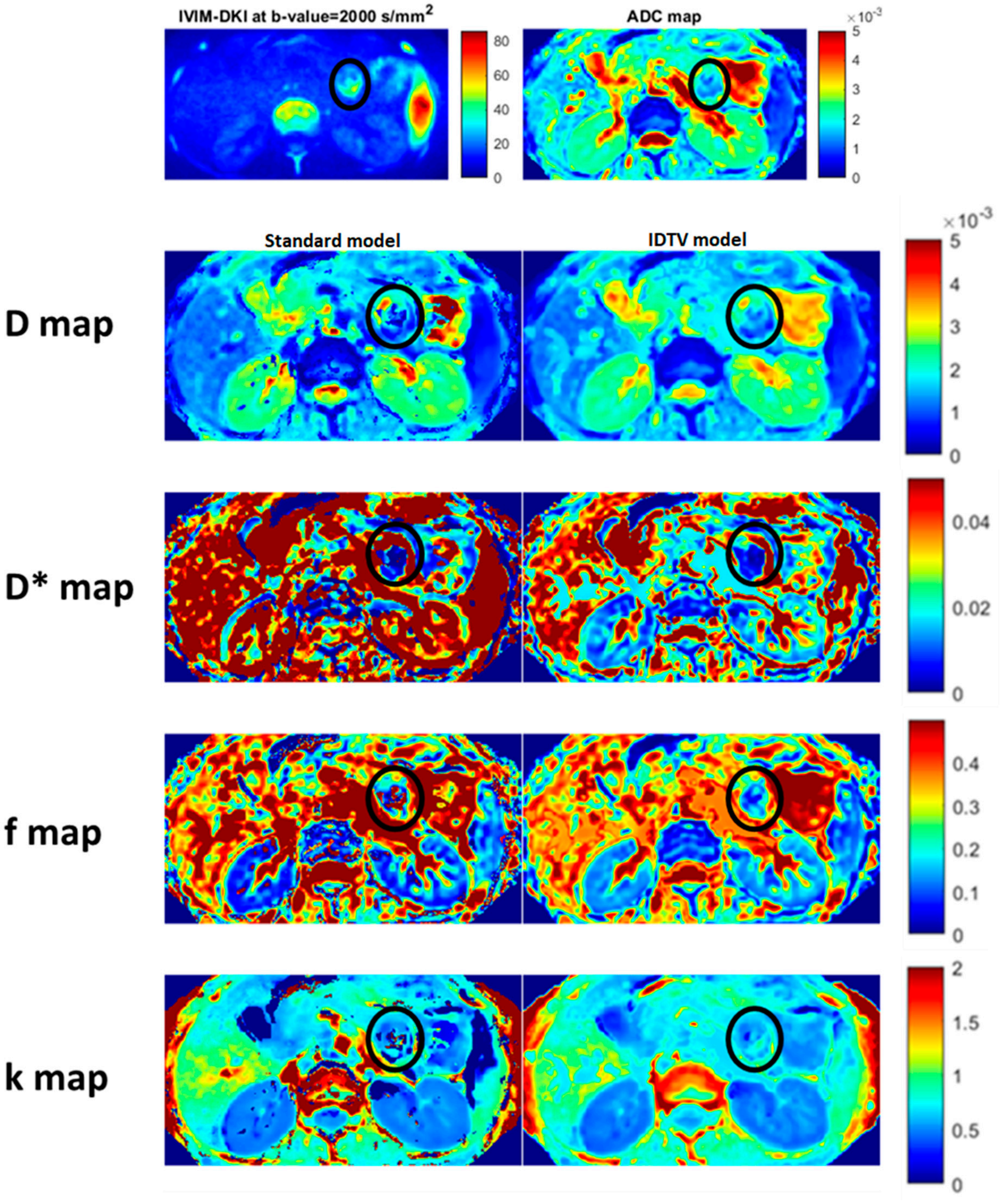

Figure S3: Representative image of a 26−years−old female patient with solid papillary epithelial neoplasm (SPEN); where qualitative comparison between parameter maps (D, D*, f and k maps) estimated using standard and IDTV model.

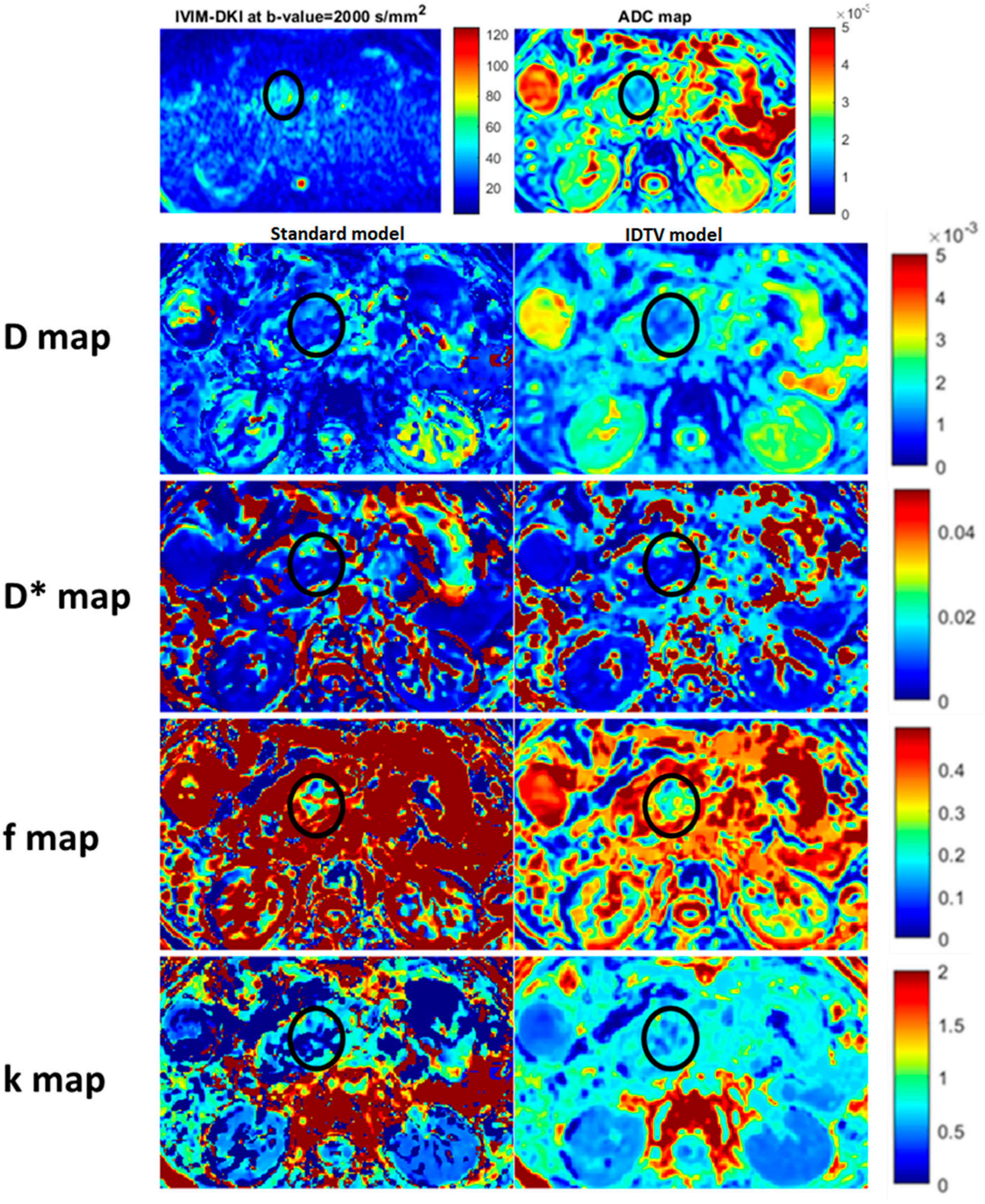

Figure S4: Representative image of a 63−year−old male patient with mass−forming chronic pancreatitis (MFCP); where qualitative comparison between parameter maps (D, D*, f and k maps) estimated using standard and IDTV model.

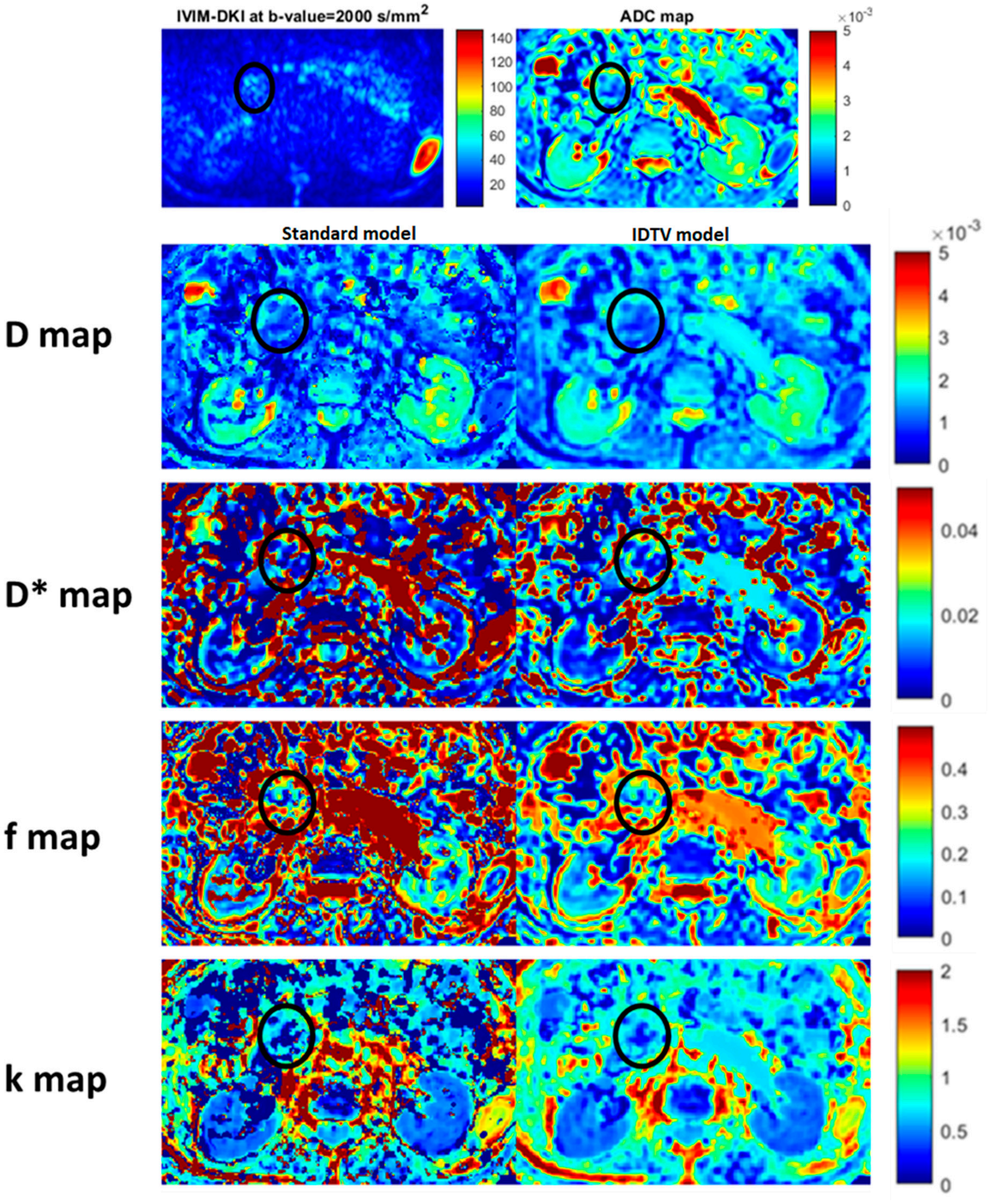

Figure S5: Representative image of a 60−years−old male patient with mass−forming chronic pancreatitis (MFCP); where qualitative comparison between parameter maps (D, D*, f and k maps) estimated using standard and IDTV model.

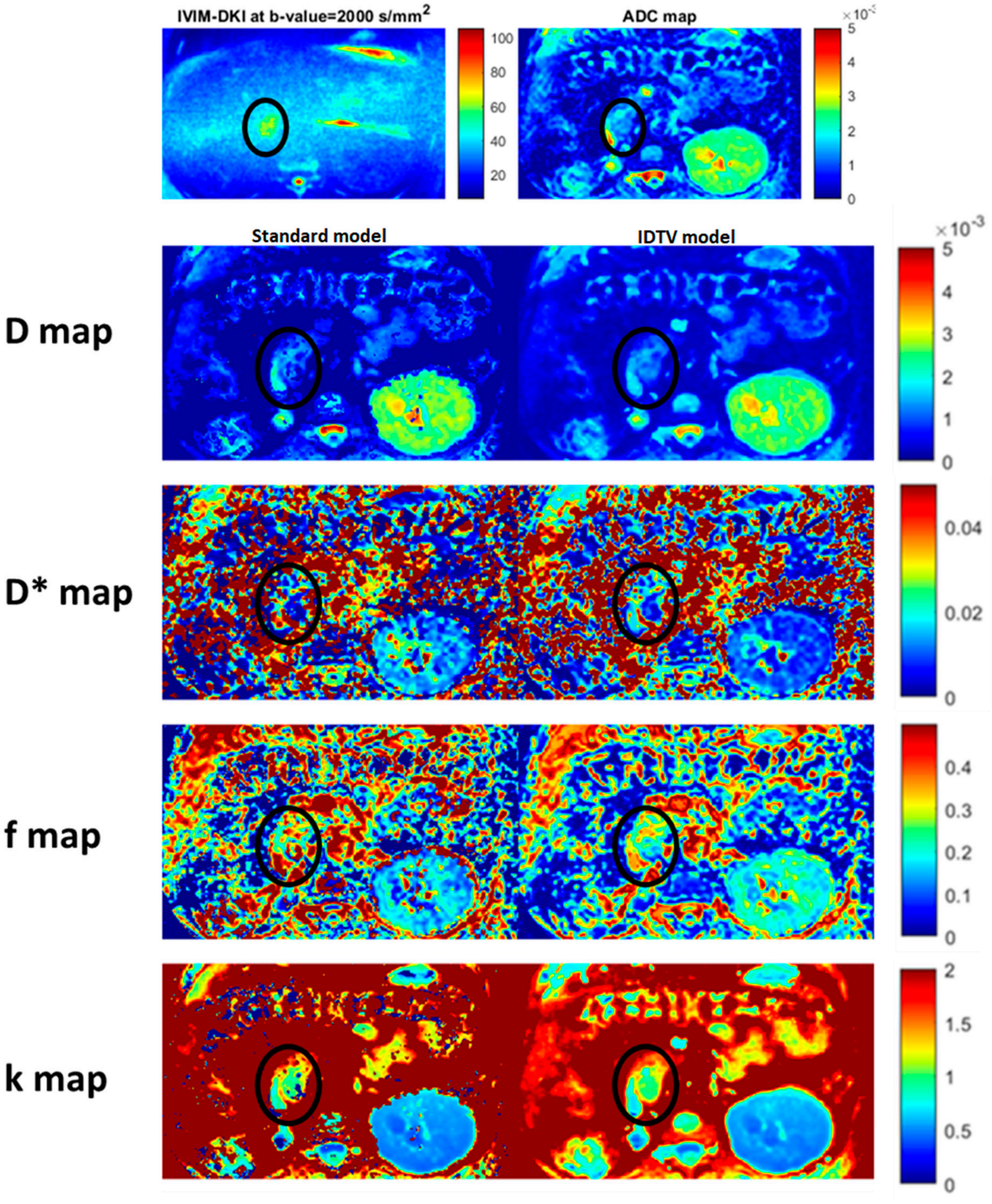

Figure S6: Representative image of a 43−years−old male patient with pancreatic neuroendocrine tumor (PNET); where qualitative comparison between parameter maps (D, D*, f and k maps) estimated using standard and IDTV model.

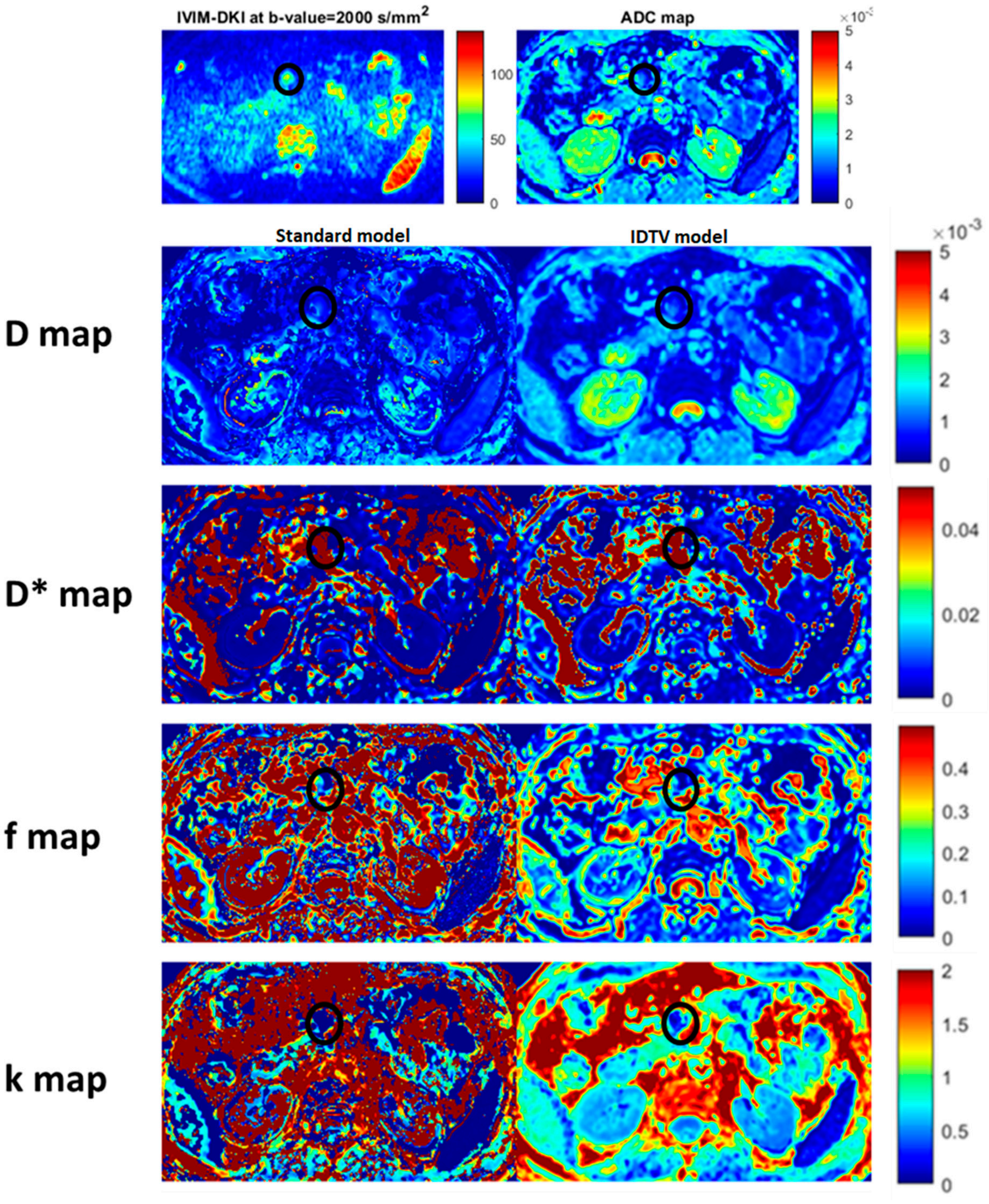

Figure S7: Representative image of a 41−years−old male patient with pancreatic neuroendocrine tumor (PNET); where qualitative comparison between parameter maps (D, D*, f and k maps) estimated using standard and IDTV model.

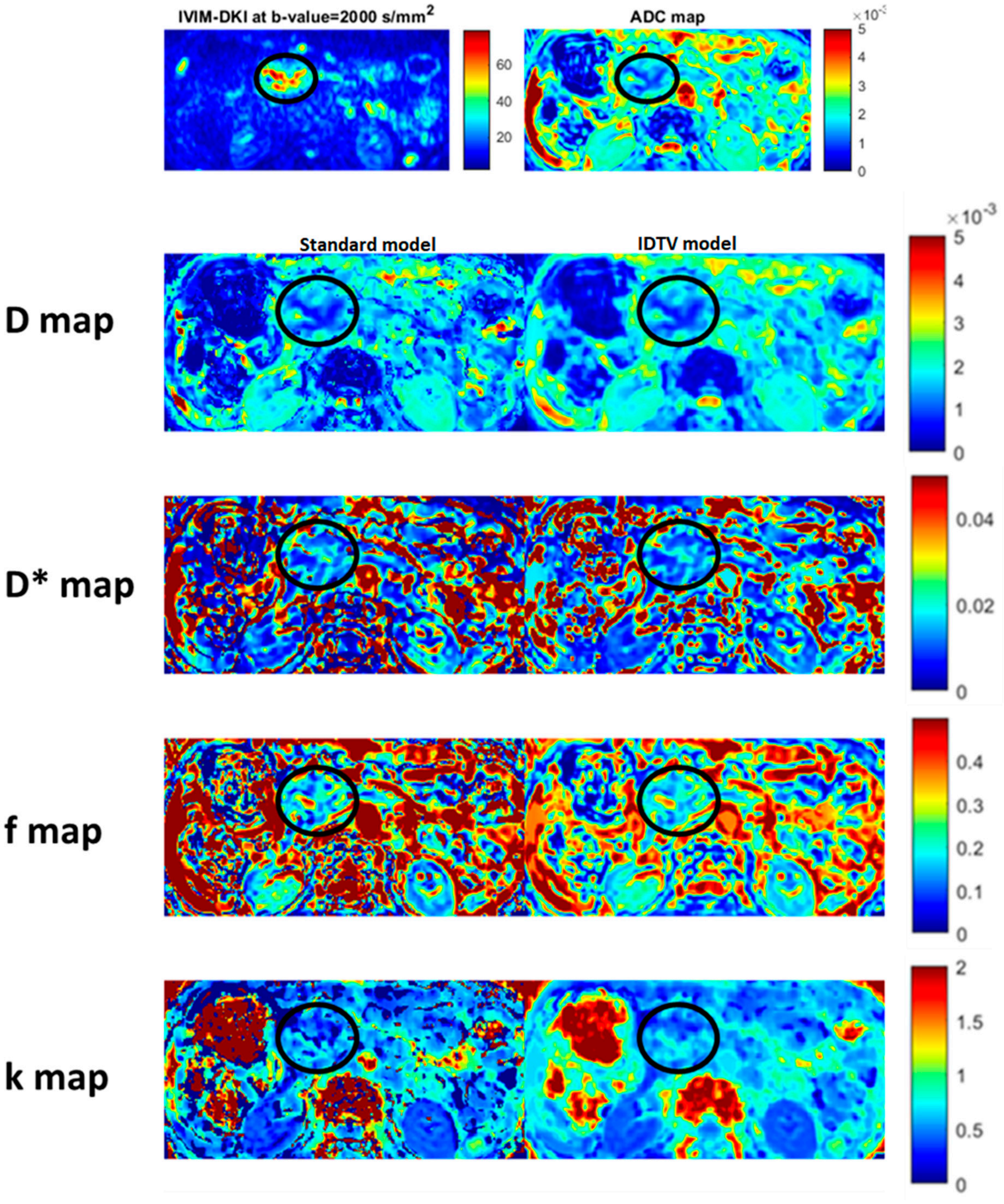

Figure S8: Representative image of a 33−years−old male patient with pancreatic ductal adenocarcinomas (PDAC); where qualitative comparison between parameter maps (D, D*, f and k maps) estimated using standard and IDTV model.

Table S4: Machine learning−based classification performance of all texture features (30 features) and top ten features selected from ADC, combined and individual IVIM−DKI parameters, and ADC combined with IVIM−DKI parameters to classify PDAC from non−PDAC pancreatic masses using decision tree and ensemble.

PDAC vs. non−PDAC		Features selected		Accuracy %	Precision %	Specificity %	F1 score %	AUC	Classification Error %
ADC	All feats (30 features)	All features	DT	62.7±3.3	61.6±2	69.5±2.8	56.8±2.8	0.69±0.05	37.3±1.9
			Ens	90±1.2	90±1.2	91.4±1	89.8±1.1	0.97±0.02	9.9±1.2
	Chi−square (Top 10 features)	f27, f30, f14, f6, f5, f4, f18, f7, f28, f9	DT	68.6±2.1	68±1.7	72.2±1.8	66.4±1.9	0.73±0.04	31.4±1.7
			Ens	90.8±0.8	90.9±0.9	92.2±0.6	90.7±0.8	0.97±0.02	9.1±0.8
IVIM−DKI	All feats (30*4 features)	All features	DT	63.7±1.2	62.9±1.3	65.5±1.1	61.4±1.8	0.7±0.05	37±1.3
			Ens	89.8±0.9	89.8±1	91.2±0.7	89.6±0.8	0.96±0.02	10.2±0.9
	Chi−square (Top 10 features)	f_parameter_f21, D*_parameter_f29, f_parameter_f13, f_parameter_f16, D*_parameter_f5, f_parameter_f9, f_parameter_f29, k_parameter_f12, k_parameter_f9, D_parameter_f9	DT	74±1.2	74.5±1	77.2±0.9	73.2±1.3	0.8±0.03	25.9±1
			Ens	85.2±0.7	85.2±0.8	87±0.7	84.9±0.7	0.94±0.02	14.8±0.7
ADC with IVIM−DKI	All feats (30*5 features)	All features	DT	91±0.8	91±0.8	92.4±0.5	90.8±0.7	0.91±0.04	8.9±0.7
			Ens	84.2±0.9	84.3±0.9	86.2±1	83.9±1	0.92±0.03	15.7±0.9
	Chi−square (Top 10 features)	f_parameter_f21, D*_parameter_f29, f_parameter_	DT	74±1.2	74.5±1	77.2±0.9	73.2±1.3	0.8±0.03	25.9±1
			Ens	85.2±0.7	85.2±0.8	87±0.7	84.9±0.7	0.94±0.02	14.8±0.7

		f13, f_parameter_f16, D*_parameter_f5, f_parameter_f9, f_parameter_f29, k_parameter_f12, k_parameter_f9, D_parameter_f9							
D	All feats (30 features)	All features	DT	58.7±1.9	56.7±1.5	71.8±3.8	47.8±2.6	0.56±0.06	41.8±1.5
			Ens	89.8±1.6	89.8±1.6	91.4±1.3	89.6±1.5	0.96±0.03	10.1±1.6
	Chi−square (Top 10 features)	f9, f1, f27, f25, f10, f17, f19, f13, f16, f24	DT	65.4±1.6	64.5±0.9	70.2±1.1	61.6±1.2	0.72±0.05	34.6±0.8
			Ens	91.8±0.9	91.8±0.9	93±0.8	91.6±0.8	0.97±0.02	8.2±0.9
D*	All feats (30 features)	All features	DT	62.1±2.4	60.7±0.8	71±0.7	55±2	0.59±0.04	38±0.6
			Ens	75.1±1.1	74.8±0.8	77.5±0.7	74±1	0.83±0.05	25±0.8
	Chi−square (Top 10 features)	f29, f5, f22, f27, f26, f25, f30, f11, f2, f1	DT	63.4±1.4	61.8±1.9	70.7±2.9	57.2±3	0.67±0.05	37.1±1.7
			Ens	69.4±1.6	68.9±1.3	73.3±1.4	67±1.5	0.78±0.05	30.7±1.2
f	All feats (30 features)	All features	DT	72.5±3.2	72.3±3	74.9±2.9	71.2±3.3	0.82±0.03	27.9±3.2
			Ens	77.4±1.2	77.6±1.3	79.5±1.1	76.9±1.2	0.85±0.02	22.5±1.2
	Chi−square (Top 10 features)	f21, f13, f16, f9, f29, f12, f2, f1, f23, f7	DT	70.4±1.9	70.8±1.8	73.4±1.7	69.4±2	0.8±0.03	29.5±1.8
			Ens	75.2±3	75.3±3.1	77.5±2.8	74.4±3.3	0.84±0.02	24.9±3.1
k	All feats (30 features)	All features	DT	68±2.1	67.1±2.1	70.6±2.8	65.9±2.3	0.72±0.04	32.7±2
			Ens	76.6±1.1	76.6±1.1	78.6±1.2	76±1	0.84±0.04	23.4±1.1
	Chi−square (Top 10	f12, f9, f18, f20, f1, f4, f22, f28, f2, f19	DT	67.9±1.5	67.8±1.8	69.8±2.3	66.7±1.8	0.74±0.03	32.4±1.7
			Ens	76.5±2.1	76.7±1.9	79±2.1	75.9±2.1	0.84±0.03	23.5±1.9

features<br>)	

DT: Decision tree; Ens: Ensemble; PDAC: Pancreatic ductal adenocarcinoma; AUC: Area under curve; ADC: Apparent diffusion coefficient; D: Diffusion coefficient; D*: Pseudo−diffusion coefficient; f: Perfusion fraction; Kurtosis; IVIM−DKI: Intravoxel Incoherent motion−diffusion kurtosis imaging;
